# A single photoreceptor splits perception and entrainment by cotransmission

**DOI:** 10.1038/s41586-023-06681-6

**Published:** 2023-10-25

**Authors:** Na Xiao, Shuang Xu, Ze-Kai Li, Min Tang, Renbo Mao, Tian Yang, Si-Xing Ma, Peng-Hao Wang, Meng-Tong Li, Ajay Sunilkumar, François Rouyer, Li-Hui Cao, Dong-Gen Luo

**Affiliations:** 1 State Key Laboratory of Membrane Biology, School of Life Sciences, Peking University, Beijing, China; 2 IDG/McGovern Institute for Brain Research, Peking University, Beijing, China; 3 Peking-Tsinghua Center for Life Sciences, Academy for Advanced Interdisciplinary Studies, Peking University, Beijing, China; 4 School of Life Sciences, Peking University, Beijing, China; 5 Center for Quantitative Biology, Academy for Advanced Interdisciplinary Studies, Peking University, Beijing, China; 6 Institut des Neurosciences Paris-Saclay, Université Paris-Sud, Université Paris-Saclay, CNRS, Gif-sur-Yvette, France; 7 School of Basic Medical Sciences, Beijing Key Laboratory of Neural Regeneration and Repair, Capital Medical University, Beijing, China; 8 Chinese Institute for Brain Research, Beijing, China; 9 Present Address: Department of Psychiatry, University of Texas Southwestern Medical Center, Dallas, TX USA; 10 Present Address: Zuckerman Mind Brain and Behavior Institute, Columbia University, New York, NY USA

**Keywords:** Circadian regulation, Sensory processing, Neurotransmitters

## Abstract

Vision enables both image-forming perception, driven by a contrast-based pathway, and unconscious non-image-forming circadian photoentrainment, driven by an irradiance-based pathway^1,2^. Although two distinct photoreceptor populations are specialized for each visual task^3–6^, image-forming photoreceptors can additionally contribute to photoentrainment of the circadian clock in different species^7–15^. However, it is unknown how the image-forming photoreceptor pathway can functionally implement the segregation of irradiance signals required for circadian photoentrainment from contrast signals required for image perception. Here we report that the *Drosophila* R8 photoreceptor separates image-forming and irradiance signals by co-transmitting two neurotransmitters, histamine and acetylcholine. This segregation is further established postsynaptically by histamine-receptor-expressing unicolumnar retinotopic neurons and acetylcholine-receptor-expressing multicolumnar integration neurons. The acetylcholine transmission from R8 photoreceptors is sustained by an autocrine negative feedback of the cotransmitted histamine during the light phase of light–dark cycles. At the behavioural level, elimination of histamine and acetylcholine transmission impairs R8-driven motion detection and circadian photoentrainment, respectively. Thus, a single type of photoreceptor can achieve the dichotomy of visual perception and circadian photoentrainment as early as the first visual synapses, revealing a simple yet robust mechanism to segregate and translate distinct sensory features into different animal behaviours.

## Abstract (web summary)

The *Drosophila* R8 photoreceptor separates signals for image perception and circadian photoentrainment by co-releasing histamine and acetylcholine, and this segregation is further established in the postsynaptic circuitry in the medulla.

## Main

The principle that irradiance signals for circadian photoentrainment can be generated by the conventional image-forming visual pathway is conserved in different species^7–15^. In mammals, vision begins with light detection by retinal rod and cone photoreceptors^16^, and sacrifices the absolute irradiance information to extract local contrast by constructing centre-surround antagonistic receptive fields in most bipolar cells^17^. Interestingly, bipolar cells also transmit irradiance information from rods and cones to intrinsically photosensitive retinal ganglion cells that are required for circadian photoentrainment^7,8^. Unlike conventional retinal ganglion cells responsible for image-forming vision, most intrinsically photosensitive retinal ganglion cells that photoentrain circadian clocks do not exhibit centre-surround antagonistic receptive fields even for rod and cone inputs^18–20^, consistent with their role in coding overall irradiance instead of contrast. However, it is unclear whether and how conventional rod and cone circuits upstream of intrinsically photosensitive retinal ganglion cells can generate irradiance signals and separate them from their image-forming signals. The circadian clock in the *Drosophila* brain also receives irradiance signals from conventional retinal photoreceptors^9–15^ but, like in mammals, the underlying neural mechanisms that generate and segregate irradiance signals remain unknown. Here we identify a mechanism that enables such segregation in the *Drosophila* visual system. A single type of retinal photoreceptor cell separates visual perception and circadian photoentrainment by co-transmitting two neurotransmitters at the first visual synapse, exemplifying a simple yet robust solution to the retina’s multi-tasking needs.

## Histamine-independent irradiance signals

**Fig. 1:**
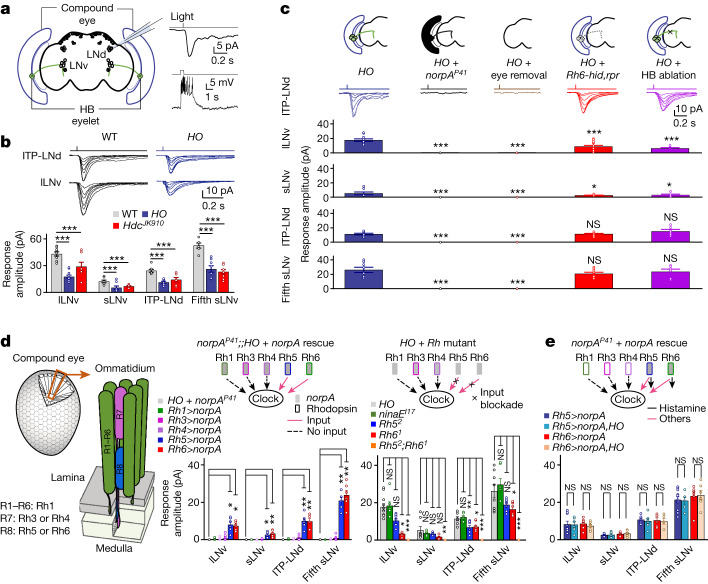
Histamine-independent irradiance signals. **a**, Left: schematic of patch-clamp recording from a clock neuron (ITP-LNd); right: representative current (top: voltage clamp) and voltage (bottom: current clamp) responses to light stimuli (470 nm, 2.51 × 10^7^ photons μm^−2^ s^−1^, top: 2 ms, bottom: 500 ms). The timing of light stimulation is indicated at the top of the response traces. Genotypes are listed in Supplementary Table 1. **b**, Histamine-independent responses of clock neurons. Top: representative light responses (470 nm, 2 ms, intensities of 0.01, 0.04, 0.20, 0.41, 0.81, 1.93 and 2.80 × 10^7^ photons μm^−2^ s^−1^). WT, wild type. Bottom: pooled saturated response amplitudes. **c**, Histamine-independent inputs require PLC signalling in compound eyes. Top: schematic of eye input manipulation; middle: representative responses of ITP-LNd in *HO* flies, *norpA*^*P41*^;;*HO* flies, *HO* flies with eyes removed, *Rh6-hid,rpr*;*HO* flies and *HO* flies with HB eyelet axons laser ablated; bottom: pooled saturated response amplitudes. Light: 470 nm, 2 ms, intensities of 0.01, 0.04, 0.20, 0.41, 0.81, 1.93 and 2.80 × 10^7^ photons μm^−2^ s^−1^. **d**, Histamine-independent signals from R8 photoreceptors. Left: schematic of a compound eye with approximately 750 ommatidia, with each ommatidium containing 8 photoreceptors (R1–R8) that express different rhodopsins (Rh1, Rh3–Rh6); middle: histamine-independent responses in *norpA*^*P41*^;;*HO* mutant flies with *norpA* rescued in different photoreceptors (with HB eyelet axons laser ablated); right: histamine-independent responses in *HO* flies with mutations in different rhodopsins (with HB eyelet axons laser ablated). **e**, R8 photoreceptors do not use histamine to transmit irradiance signals. Top: schematic of photoreceptor signalling; bottom: pooled saturated response amplitudes of R8 photoreceptors of *norpA*^*P41*^ flies with *norpA* rescued in pR8 or yR8 photoreceptors (with or without *HO* mutants; with HB eyelet axons laser ablated). Light in **d**,**e**: 470 nm, 2 ms, 2.80 × 10^7^ photons μm^−2^ s^−1^. Pooled data are shown as mean ± s.e.m. *P < 0.05; **P < 0.01; ***P < 0.001; NS, not significant. Statistical analysis is summarized in Supplementary Table 2.

In *Drosophila*, there are several pathways for photoentrainment. First, a blue-light-sensitive cryptochrome expressed in clock neurons can photoentrain flies in a cell-autonomous manner^6^. Second, visual inputs from eye structures can photoentrain flies in a NorpA-dependent manner^11,13,15,21^. Third, flies can also photoentrain in a NorpA- and cryptochrome-independent manner^11,14,22^. *Drosophila* have three types of eye structure: ocelli, Hofbauer–Buchner (HB) eyelets and compound eyes^23^, the last of which generate signals for both image-forming vision and circadian photoentrainment^9–15^. As, in addition to the canonical neurotransmitter histamine used for image-forming vision^24^, acetylcholine (ACh) has recently been implicated in a subgroup of compound eye photoreceptors^9,24–28^, we examined whether light-induced depolarization in circadian clock neurons^12^ depends on the canonical histamine signalling or other neurotransmitter signalling from photoreceptors (Fig. 1a).

**Extended Data Fig. 1:**
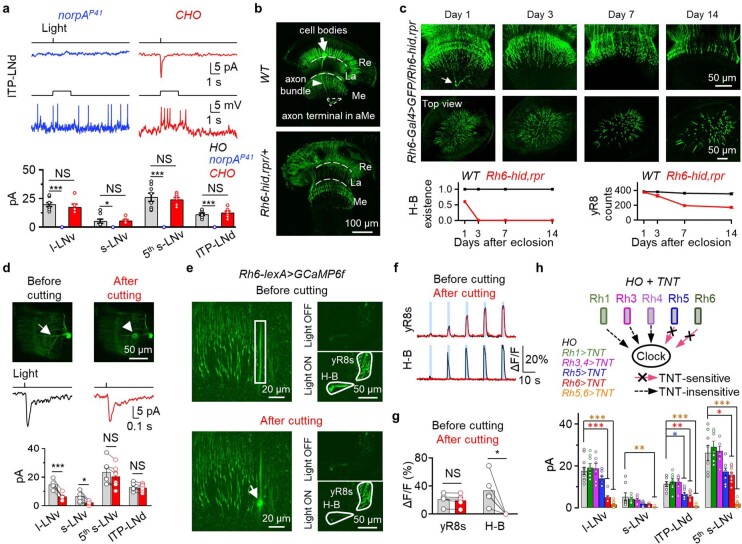
R8-mediated irradiance signals. **a**, PLC-dependent, but histamine- and cryptochrome-independent light responses. **Top**, Representative light-triggered currents of ITP-LNd in *norpA*^*P41*^ (left) and *CHO* (right) flies; **middle**, representative light-triggered depolarization; **bottom**, pooled data. Light: 470 nm, 5.65 × 10^7^ photons/μm^2^/s, 2 ms (top) and 2 s (middle). **b**, H-B eyelet ablation in *Rh6-hid,rpr* flies on day 3 after eclosion. **c**, Time-dependent H-B eyelet ablation by *Rh6-hid,rpr*. **Top**, H-B eyelet axons and yR8s on different days after eclosion; **middle**, top views of yR8 loss; **bottom**, probability of H-B eyelet existence (left), and pooled data of yR8 counts (right). Arrow: H-B eyelet axons. **d**, Histamine-independent light responses remained in the absence of H-B eyelets. **Top**, schematic of laser cutting H-B axons, **middle**, representative ITP-LNd responses; **bottom**, pooled data. Arrow: intact GFP-labeled H-B eyelet axons; arrowhead: a cavitation bubble associated with laser-ablation of H-B eyelet axons. Light stimulation: 470 nm, 2 ms, 5.65 × 10^7^ photons/μm^2^/s. **e**-**g**, Specificity and completeness of laser cutting H-B eyelet axons. **e**, **Top**, representative H-B eyelets and yR8s (left), and their calcium response to light stimulation (right); **bottom**, laser cutting of H-B eyelet axons (left), and representative light-triggered calcium responses. Light stimulation: 460 nm, 2 s, 1.18 × 10^6^ photons/μm^2^/s. White rectangle: axon bundle of H-B eyelets; arrow: a cavitation bubble associated with laser ablation. **f**, Representative calcium responses of yR8s (top) and H-B eyelets (bottom). Light stimulation: 460 nm, 2 s, intensities of 0.03, 0.30, 0.97, 1.18, and 2.36 × 10^6^ photons/μm^2^/s. Blue bars: timing of light stimulation. **g**, Pooled calcium responses of yR8s (n = 5 flies) and H-B eyelets (n = 5 flies). Light stimulation: 460 nm, 2 s, 1.18 × 10^6^ photons/μm^2^/s. **h**, Histamine-independent responses after photoreceptor silenced by TNT. Pooled data: mean ± s.e.m. *P < 0.05; **P < 0.01; ***P < 0.001; NS, not significant. Statistical analysis is summarized in Supplementary Table 2.

Unexpectedly, clock neurons (including arousal neurons^29^: large ventrolateral neurons (lLNvs); morning cells^30,31^: small ventrolateral neurons (sLNvs); and evening cells^30,31^: ion transport peptide-expressing dorsolateral neuron (ITP-LNd) and the fifth sLNv exhibited robust, although reduced, light-induced depolarization in the null mutant of the histamine-synthesizing enzyme histidine decarboxylase (HDC) *Hdc*^*JK910*^ (ref. ^32^) or null mutants of both histamine receptors in photoreceptors and glia (*HisCl1*) and second-order retinal neurons (*ort*)^9,33,34^ (*HO*; *HisCl1*^*134*^*,ort*^*1*^) (Fig. 1b). These responses were independent of cryptochrome^5,6^ as they remained in the triple-mutant flies lacking cryptochrome, HisCl1 and Ort (*CHO*; *cry*^*02*^*,HisCl1*^*134*^*,ort*^*1*^; Extended Data Fig. 1a), suggesting that clock neurons receive non-histaminergic inputs from the eyes. Furthermore, non-histaminergic inputs were completely absent in flies lacking phospholipase C (PLC; *norpA*^*P41*^) under light intensities tested in this work (Fig. 1c and Extended Data Fig. 1a), revealing their dependence on PLC-mediated canonical phototransduction. Physical removal of compound eyes and HB eyelets abolished non-histaminergic inputs to clock neurons (Fig. 1c), but these inputs remained, although at reduced levels, following genetic (Fig. 1c and Extended Data Fig. 1b,c) or laser (Fig. 1c and Extended Data Fig. 1d–g) ablation of HB eyelets. Thus, compound eyes can generate non-histaminergic signals that excite clock neurons.

To investigate which photoreceptor subtype generates these non-histaminergic signals in compound eyes, we genetically manipulated the functions of individual photoreceptor subtypes in flies with HB eyelets laser ablated (Fig. 1d). Histamine-independent responses of clock neurons were intact after transmission blockade in R1–R7 photoreceptors expressing Rh1, Rh3 or Rh4, but reduced following blockade in Rh5-expressing pale R8 (pR8) or Rh6-expressing yellow R8 (yR8) photoreceptors^35^ (Extended Data Fig. 1h). Furthermore, *norpA* rescue in pR8 or yR8 photoreceptors partially restored histamine-independent responses in *norpA*^*P41*^;;*HO* flies with HB eyelets laser ablated (Fig. 1d), confirming that R8 photoreceptors provide non-histaminergic inputs to the circadian clock. Finally, these responses were completely lost when both pR8 and yR8 photoreceptors were blocked (Extended Data Fig. 1h) or when both Rh5 and Rh6 were mutated (Fig. 1d), demonstrating their origin exclusively from R8 photoreceptors. As R8-mediated responses to brief pulses of light were comparable in the presence or absence of histamine receptors in *norpA*^*P41*^ flies with HB eyelets laser ablated (Fig. 1e), we concluded that although histamine is the canonical neurotransmitter of the compound eyes, R8 photoreceptors do not use it to transmit irradiance signals to clock neurons.

## R8 photoreceptors release two neurotransmitters

**Fig. 2:**
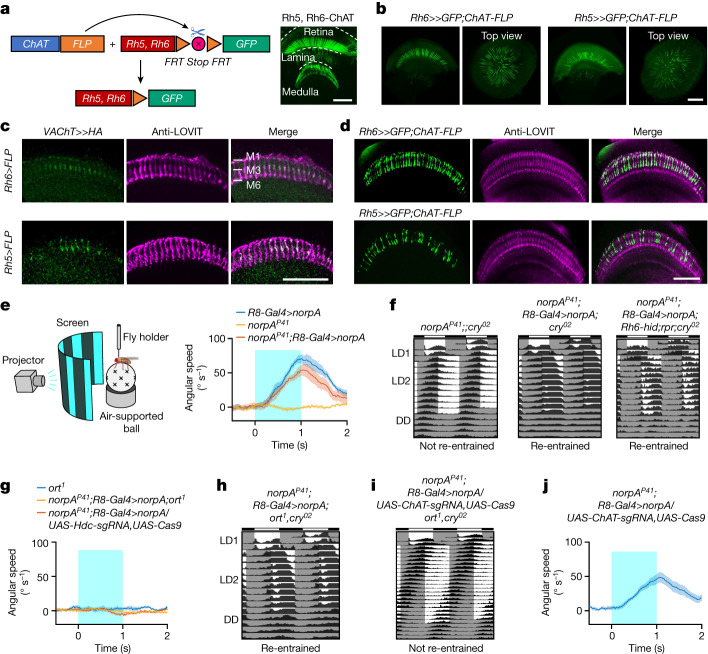
R8 photoreceptors use histamine for motion detection and ACh for circadian photoentrainment. **a**, R8 photoreceptors contain ACh. Left: schematic of genetic intersection; right: ChAT in R8 photoreceptors (*Rh5-Gal4* + *Rh6-Gal4*). Scale bar, 100 μm. **b**, ACh in pR8 (*Rh5-Gal4*) and yR8 (*Rh6-Gal4*) photoreceptors. Scale bar, 100 μm. **c**, Single R8 photoreceptors express both VAChT and LOVIT. Scale bar, 50 μm. **d**, Single R8 photoreceptors express both ChAT and LOVIT. Scale bar, 50 μm. **e**, Motion detection by R8 photoreceptors. Left: schematic of behavioural motion detection; right: pooled angular speed in *R8-Gal4* (*Rh5-Gal4,Rh6-Gal4*)*>norpA* (*n* = 12), *norpA*^*P41*^ (*n* = 18) and *norpA*^*P41*^*;R8-Gal4>norpA* (*n* = 13) flies. **f**, Circadian photoentrainment by R8 photoreceptors. Average actograms of *norpA*^*P41*^;;*cry*^*02*^ flies (left: *n* = 102), flies with only R8 photoreceptors and HB eyelets in dim-light detection (middle: *n* = 93) and flies with only R8 photoreceptors in dim-light detection (with HB eyelets ablated by *Rh6-hid,rpr*; right: *n* = 73). **g**,**h**, *ort* is indispensable for R8-mediated motion detection but not circadian photoentrainment. **g**, Motion detection in *ort* mutants (*n* = 13), *ort* mutants with functional R8 photoreceptors (*n* = 11) or mutants with *Hdc*-knockout in R8 photoreceptors (*n* = 11). **h**, Average actograms following genetic ablation of *ort* in *norpA*^*P41*^;;*cry*^*02*^ flies with *norpA* rescued in R8 photoreceptors (*n* = 91). **i**,**j**, ACh signalling is required for R8-mediated circadian photoentrainment but not motion detection. **i**, Average actograms following genetic ablation of both *ort* and *ChAT* in *norpA*^*P41*^;;*cry*^*02*^ flies with *norpA* rescued in R8 photoreceptors (*n* = 74). **j**, Pooled angular speed for motion detection by R8 photoreceptors after *ChAT* knockout in *norpA*^*P41*^ flies with *norpA* rescued in R8 photoreceptors (*n* = 12). **e**,**g**,**j**, visual moving bars: wave width of 30°, angular velocity of 180° s^−1^, contrast of 100%, duration of 1 s. Data are represented as mean (solid line) ± s.e.m. (shading). **f**,**h**,**i**, LD1: 200 lux (white light) together with 25 °C/18 °C temperature cycles; LD2: 0.05 lux (white light) at 25 °C; DD: 25 °C. Low-intensity light (0.05 lux) in LD2 cycles is used to examine NorpA-dependent re-entrainment.

**Extended Data Fig. 2:**
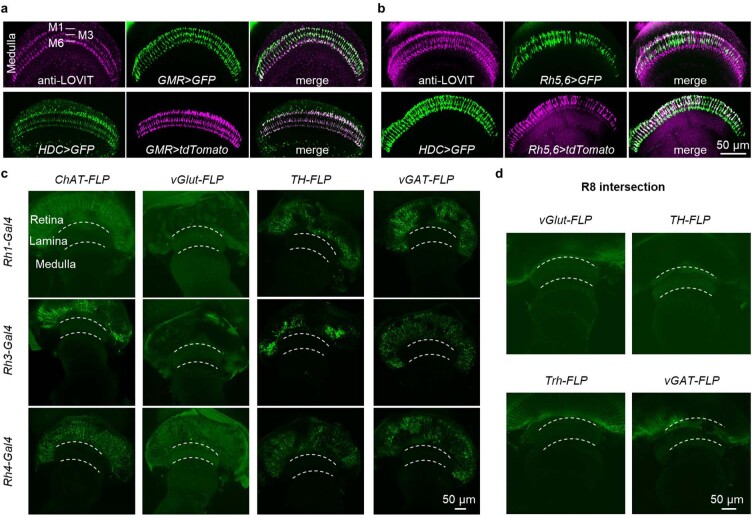
R8s contain histamine. **a**, Histamine in compound eyes. **Top**, anti-LOVIT staining in the medulla; **bottom**, GFP expression by *Hdc-Gal4* in the medulla. R8s terminate in the medullar layers of M1-M3, and R7s in M1-M6. *GMR-LexA* labeled all eye photoreceptors. **b**, R8s contain histamine. **Top**, anti-LOVIT staining; **bottom**, HDC expression. **c**, No ACh, glutamate, dopamine, and GABA in R1-R7 photoreceptors. **d**, No glutamate, dopamine, serotonin, and GABA in R8s.

Histamine is the known canonical neurotransmitter used by compound eyes^24^; we confirmed this by observing the vesicular histamine transporter LOVIT (loss of visual transmission)^36^ in the medulla (Extended Data Fig. 2a), where R8 axons terminate. In addition, we observed a similar expression pattern for HDC (Extended Data Fig. 2a). Double labelling confirmed that R8 photoreceptors express both LOVIT and HDC (Extended Data Fig. 2b), and thus have the ability to use histaminergic neurotransmission. We therefore sought to identify the neurotransmitter responsible for their non-histaminergic signals by utilizing the recently developed chemoconnectomics tool^37^ (Fig. 2a). Genetic intersection revealed that R8 photoreceptors express the ACh-synthesizing enzyme ChAT (Fig. 2a,b), as well as the vesicular ACh transporter VAChT (Fig. 2c), consistent with the previous RNA-sequencing results^26^. Notably, VAChT- and ChAT-expressed R8 photoreceptors were also immunopositive for LOVIT (Fig. 2c,d), suggesting that single R8 photoreceptors release both histamine and ACh during neurotransmission. By contrast, ChAT was not expressed in R1–R7 photoreceptors (Extended Data Fig. 2c), and other transmitters, including GABA, glutamate, serotonin and dopamine, were absent in R8 photoreceptors (Extended Data Fig. 2d).

**Extended Data Fig. 3:**
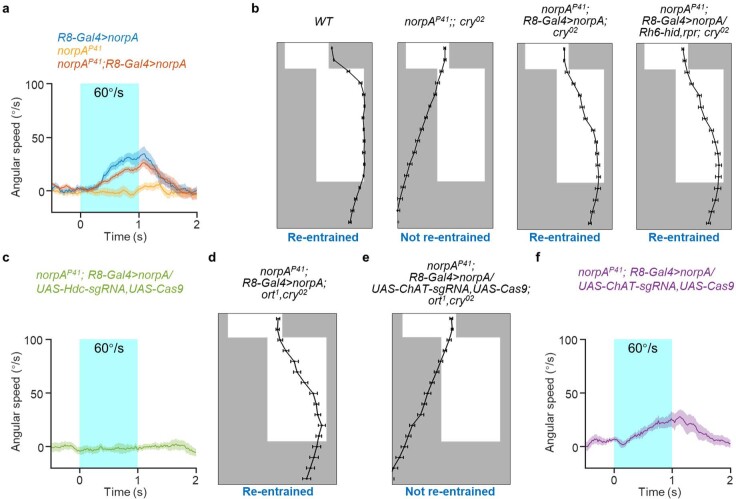
R8s use histamine for motion detection and ACh for circadian photoentrainment. **a**, Motion detection by R8s to slow (60°/s) visual stimuli. Pooled angular speed in *norpA*^*P41*^ (n = 10), *R8-Gal4 (Rh5-Gal4,Rh6-Gal4)>norpA* (n = 12), and *norpA*^*P41*^*; R8-Gal4>norpA* (n = 11) flies. Data are represented as mean (solid line) and s.e.m. (shading). **b**, Circadian photoentrainment by R8s. Phase plots of evening peaks of wild-type flies (left, n = 33), *norpA*^*P41*^;; *cry*^*02*^ mutant flies (second panel from left, n = 102), the flies with only R8s and H-B eyelets in dim-light detection (third panel from left, n = 93), and the flies with only R8s in dim-light detection (with H-B eyelets genetically ablated by *Rh6-hid,rpr*) (right, n = 73). **c**, Motion detection by R8s depends on histamine. Pooled angular speed of flies with *Hdc*-knockout in R8s (n = 11). **d** and **e**, Phase plots of evening peaks following genetic loss of *ort* (**d**, n = 91), or loss of both *ort* and *ChAT* (**e**, n = 74) in *norpA*^*P41*^;; *cry*^*02*^ flies with norpA rescued in R8s. **f**, R8-mediated motion detection does not depend on ACh from R8s. Pooled angular speed for R8-mediated motion detection after *ChAT* knockout in *norpA*^*P41*^ flies with norpA rescued in R8s (n = 9). Visual moving bars in **a**, **c**, and **f**: wave width of 30°, angular velocity of 60°/s, contrast of 100%, duration of 1 s. Pooled data: mean ± s.e.m.

**Extended Data Fig. 4:**
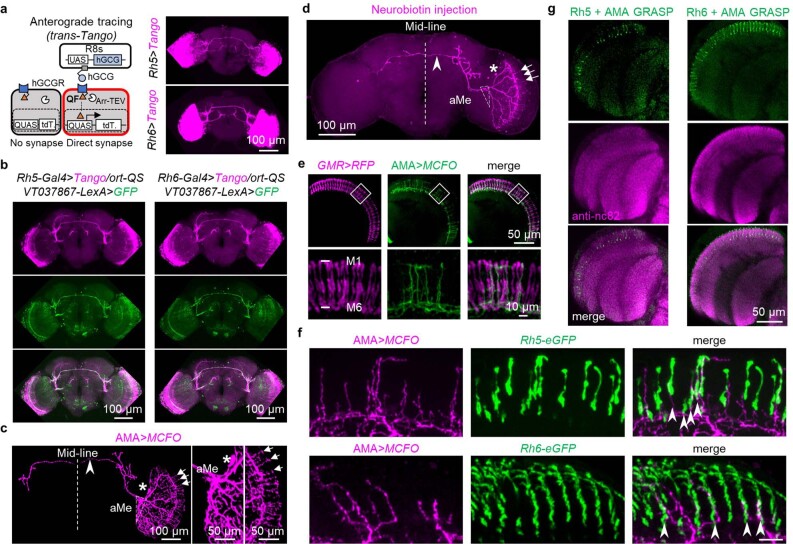
Morphological features of AMA neurons. **a**, Postsynaptic candidates of R8s. **Left**, schematic of *trans*-Tango labeling; **right**, postsynaptic neurons of pR8s (*Rh5-Gal4*) and yR8s (*Rh6-Gal4*). **b**, *VT037867-*labled neurons overlap with R8s’ postsynaptic neurons that do not express *ort*. **c**, Single MCFO-labeled AMA neuron innervates the aMe, arborizes in multiple medullar visual columns, and exhibits an arcuate dorsal commissure. **Left**, an overview of the entire brain; **middle**, an expanded view of the aMe region; **right**, an expanded view of columnar arborization. Asterisk: cell body; dashed triangle: aMe; arrows: arborization in medulla columns; arrowhead: arcuate dorsal commissure. **d**, Single-cell morphology of the recorded AMA neurons. Single-cell morphology of a recorded AMA neuron, revealed by neurobiotin injection via the recording pipette after whole-cell recordings. Asterisk: cell body; dashed triangle: aMe; arrows: multicolumnar arborization; arrowhead: arcuate dorsal commissure. **e**, Multicolumnar arborization of AMA neurons. **Top**, RFP-labeled photoreceptors and a MCFO-labeled AMA neuron; **bottom**, an expanded view of the dashed boxes in top panels. **f**, Color inputs to AMA neurons. AMA neurons innervate pR8 columns (Rh5-eGFP, top), and yR8 columns (Rh6-eGFP, bottom). Arrowheads: overlap between an AMA neuron and R8s. Scale bar: 10 µm. **g**, Synapses between R8s and AMA neurons. Single-section image of GRASP signals between AMA neurons and pR8s (**left**) and yR8s (**right**).

As R8 photoreceptors have been implicated in both colour vision^34,38,39^ and circadian photoreception^9,10,14^, we wondered whether their two transmitters carry out distinct roles and investigated these roles with behavioural tests. Owing to the lack of a robust assay for colour vision^40^ and the interactions between motion detection and colour vision^41^, we monitored fly behavioural responses to motion stimuli^42^. By rescuing *norpA* in R8 photoreceptors of *norpA*^*P41*^ mutant flies, we could study the specific role of R8 photoreceptors in motion detection (Fig. 2e) and circadian photoentrainment by monitoring locomotion activity when HB eyelets were genetically ablated (Fig. 2f). We found that *norpA* rescue in R8 photoreceptors restored the ability of *norpA*^*P41*^ mutant flies to track moving bars (Fig. 2e and Extended Data Fig. 3a) and re-entrain to phase shifts of dim light–dark (LD) cycles (Fig. 2f and Extended Data Fig. 3b). Genetically disabling the histamine-synthesizing enzyme HDC in R8 photoreceptors or the histamine receptor *ort* abolished R8-mediated motion detection (Fig. 2g and Extended Data Fig. 3c) and the loss of *ort* did not abolish circadian photoentrainment (Fig. 2h and Extended Data Fig. 4d). Conversely, *ChAT* knockout in R8 photoreceptors abolished circadian photoentrainment (Fig. 2i and Extended Data Fig. 3e) but not motion detection (Fig. 2j and Extended Data Fig. 3f). We therefore concluded that R8 photoreceptors use histamine and ACh to drive motion detection and circadian photoentrainment, respectively. Owing to the difference in the experimental conditions between the electrophysiology and behaviour assays, it was not possible to apply the same light stimuli in the two sets of experiments. Thus, it is not possible to directly translate the electrical responses in clock neurons to behaviour. For example, light triggered an electrical response in clock neurons of *norpA*^*P41*^ mutant flies with *norpA* rescue by *Rh5-Gal4* (Fig. 1d,e) but did not photoentrain the same flies^15^.

## Distinct targets by ACh and histamine

**Fig. 3:**
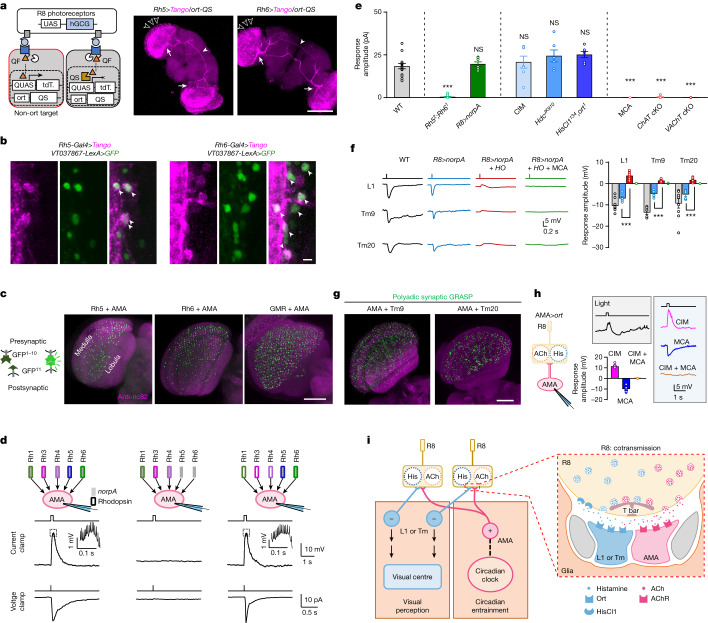
ACh and histamine act on distinct neurons. **a**, *ort*-independent postsynaptic neurons. Left: schematic of *trans*-Tango tracing with *ort-QS* excluding QF-driven tdTomato (tdT.) expression in *ort*-expressing neurons; right: *ort*-independent postsynaptic neurons of pR8 and yR8 photoreceptors. Open arrowheads mark multicolumnar arborization, arrows mark accessory medulla innervation, and filled arrowheads mark the arcuate commissure. Scale bar, 100 μm. **b**, VT037867-labelled neurons overlap with *ort*-independent postsynaptic neurons of pR8 (left) and yR8 (right) photoreceptors. Arrowheads mark co-labelled cells. Scale bar, 5 μm. **c**, Connections between R8 photoreceptors and AMA neurons. Left: schematic of GRASP labelling; right: GRASP between AMA neurons and pR8 (*Rh5-Gal4*), yR8 (*Rh6-Gal4*) or all (*GMR-Gal4*) photoreceptors. Scale bar, 50 μm. **d**, AMA neurons are excited by R8 photoreceptors. Top: schematic of photoreceptor manipulation: wild-type flies (left), *Rh5*^*2*^;*Rh6*^*1*^ flies (middle) and *norpA*^*P41*^ flies with *norpA* rescued in R8 photoreceptors (right). Middle: representative light-induced depolarization; insets represent spikes outlined by the dashed line. Bottom: representative light-induced inward current. Light: 470 nm, 5.56 × 10^7^ photons μm^−2^ s^−1^, 200 ms (current clamp) and 2 ms (voltage clamp). **e**, Light-induced saturated responses in AMA neurons. cKO, conditional knockout. **f**, Light-induced hyperpolarization of *ort*-expressing postsynaptic neurons of R8 photoreceptors. Left: representative light-induced hyperpolarization of L1, Tm9 and Tm20 neurons. Right: pooled saturated response amplitudes. **g**, AMA and Tm9 or Tm20 neurons share the same polyadic R8 synapse. **h**, Histamine-mediated responses in *ort*-expressing AMA neurons. Left: schematic of R8 inputs. Right: representative biphasic light responses in normal saline (top left); depolarization in CIM, hyperpolarization in MCA and no response in CIM + MCA (right) and pooled data (bottom left). **i**, A model of postsynaptic segregation of cotransmission. Although histamine signalling dominates in transmission from R8 photoreceptors to L1 and Tm pathways, very minor ACh signalling also exists in this pathway (**f**). Light in **d**–**f**,**h**: 470 nm, 5.56 × 10^7^ photons μm^−2^ s^−1^, duration of 2 ms (**e**,**f**) and 200 ms (**d**,**h**). Pooled data are shown as mean ± s.e.m. ***P < 0.001; NS, not significant. Statistical analysis is summarized in Supplementary Table 2.

**Extended Data Fig. 5:**
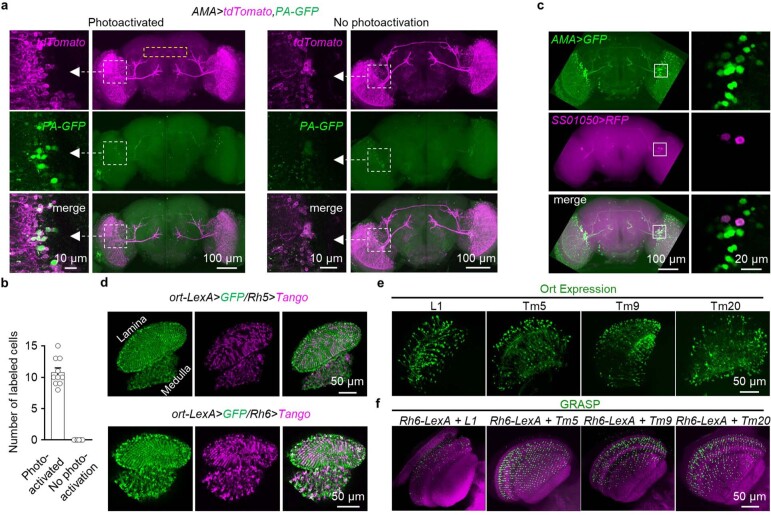
Postsynaptic neurons of R8s. **a**, PA-GFP labeling of AMA neurons postsynaptic to R8s. **Left**, tdTomato is used to identify the dorsal commissure (yellow dashed box) in *VT037867-Gal4,UAS-tdTomato,UAS-PA-GFP* flies for photoactivation (top), PA-GFP-labeled cell bodies and commissure of AMA neurons (middle), merged image (bottom); **right**, negative control of PA-GFP labeling: tdTomato-labeled dorsal commissure track in *VT037867-Gal4,UAS-tdTomato,UAS-PA-GFP* flies (top), no PA-GFP-labeling without photoactivation (middle), merged image (bottom). **b**, pooled total number of PA-GFP-labeled AMA neurons. Pooled data: mean ± s.e.m. **c**, aMe 12 belong to AMA neurons. **Left**, Co-labeling of aMe 12 (*SS01050* > *RFP*) and AMA neurons (*VT037867-LexA* > *GFP*); **right**, an expanded view of the dashed boxes. **d**, Ort-expressing neurons postsynaptic to R8s. Overlap of anterograde tdTomato labeling with *trans*-Tango and *ort-LexA* > *GFP* revealed ort-expressing postsynaptic neurons downstream of pR8s (top) and yR8s (bottom). **e**, Histamine-responsive neurons postsynaptic to R8s. GFP expression is intersected by *ort-LexA* > *FLP* and *L1, Tm5, Tm9, or Tm20-spGal4*. **f**, GRASP between R8s and L1, Tm5, Tm9, and Tm20.

To dissect the downstream circuits that support the segregation of R8-mediated visual perception from circadian photoentrainment, we labelled postsynaptic neurons of R8 photoreceptors with the anterograde trans-synaptic tracing tool *trans*-Tango^43^ (Extended Data Fig. 4a). To selectively label ACh-responsive postsynaptic neurons, we further used *ort-QS* to exclude histamine-responsive neurons by suppressing QF-driven tdTomato expression in *trans*-Tango-labelled Ort-expressing postsynaptic neurons^9^. We found one subgroup of postsynaptic multicolumnar neurons that innervate the accessory medulla, the hub that relays eye inputs to clock neurons^12^, and also show both multicolumnar arborization in the medulla and contralateral projection through a dorsal commissure with the shape of a working recurve bow (Fig. 3a). A subpopulation of extra-clock electrical oscillator (xCEO) neurons labelled by *VT037867-Gal4* (ref. ^44^) showed similar morphological features. These xCEOs overlapped with the *trans*-Tango-labelled postsynaptic Ort-independent neurons of R8 photoreceptors by sharing the same arcuate dorsal commissure (Fig. 3b and Extended Data Fig. 4b). Our single-cell morphological analysis of xCEOs by MultiColor FlpOut (MCFO) revealed their characteristic features with accessory medulla innervation, multicolumnar arborization and an arcuate dorsal commissure (Extended Data Fig. 4c), which are consistent with the single-cell morphology revealed by neurobiotin injection to a single neuron (Extended Data Fig. 4d). We named these accessory medulla-innervating, multicolumnar and arcuate neurons AMA neurons on the basis of their unique morphological features. In addition, these AMA neurons contact both pR8 and yR8 visual columns (Extended Data Fig. 4e,f). Moreover, GFP reconstitution across synaptic partners (GRASP) confirmed close contacts between R8 photoreceptors and AMA neurons in the M1–M3 layers of the medulla (Fig. 3c and Extended Data Fig. 4g), where R8 axons terminate. Furthermore, photoactivation of the photoactivatable-GFP-expressing AMA neurons specifically in the commissure track revealed a total of approximately ten pairs of AMA neurons (Extended Data Fig. 5a,b). Notably, we found that the type 12 accessory medulla neurons (aMe 12) identified as the postsynaptic neurons of R8 photoreceptors^45^ were a subgroup of AMA neurons (Extended Data Fig. 5c). To identify histamine-responsive postsynaptic neurons, we carried out double labelling with *ort-LexA*-driven GFP and R8-driven *trans*-Tango. A large population of visual neurons were co-labelled (Extended Data Fig. 5d), including L1, Tm5, Tm9 and Tm20, all known to be postsynaptic partners of R8 photoreceptors^26,38,39,45–47^. Chemoconnectomics confirmed that L1, Tm5, Tm9 and Tm20 express *ort* (Extended Data Fig. 5e), and GRASP confirmed their close contact with R8 photoreceptors (Extended Data Fig. 5f), consistent with previous reports^26,38,45^. Together, these data show that R8 photoreceptors make connections with two populations of visual neurons: multicolumnar AMA neurons receiving non-histaminergic inputs and unicolumnar L1 and Tm neurons receiving histaminergic inputs.

**Extended Data Fig. 6:**
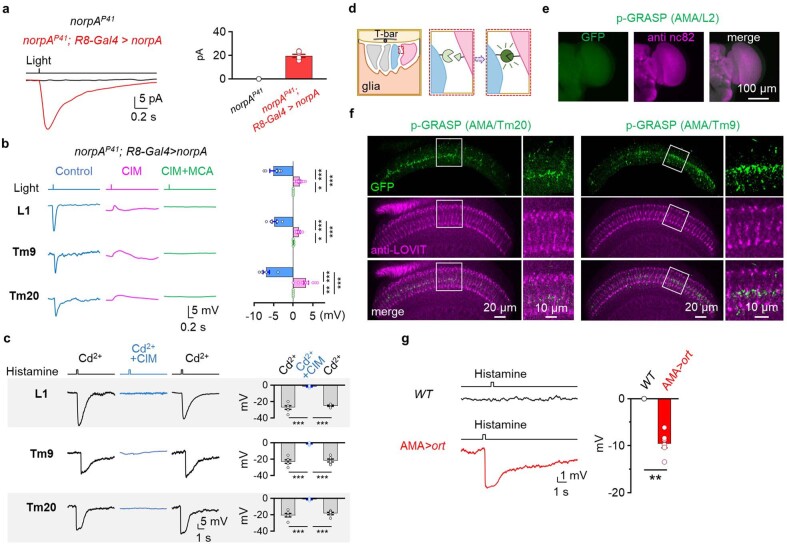
Electrical and anatomic features of postsynaptic neurons of R8s. **a**, R8-mediated light responses in AMA neurons. **Left**, Representative response of AMA neurons in *norpA*^*P41*^ flies and *norpA*^*P41*^ flies with norpA-rescued in R8s; **right**, pooled data. Light stimulation: 470 nm, 2 ms, 5.56 × 10^7^ photons/μm^2^/s. **b**, Histamine hyperpolarizes L1, Tm9 and Tm20 neurons. **Left**, representative hyperpolarization (first column), which is blocked by 2 mM CIM (second column) and recovered after wash out (third column) in the presence of Cd^2+^. **Right**, pooled data. Histamine: 1 mM, duration of 200 ms. **c**, R8-driven light responses in L1, Tm9, and Tm20 neurons require histamine. **Left**, representative light responses; **right**, pooled data. Light: 470 nm, 2 ms, 5.65 × 10^7^ photons/μm^2^/s. Note a tiny light-triggered depolarization in the absence of histamine signaling. **d**, Schematic of p-GRASP labeling. Two complementary split-GFP segments fused to ICAM5 are expressed in postsynaptic sites of two different neurons. GFP signals appear when these two neurons share the same presynaptic site. **e**, Negative control of p-GRASP. No p-GRASP signal between AMA neurons (*VT037867-LexA*) and L2 neurons (*SS00690*-*spGal4*), consistent with the no sharing of presynaptic site from the hemibrain connectome data. **f**, AMA neurons share the same polyadic R8 synapse with Tm20/Tm9. **Left**, p-GRASP between AMA neurons (*VT037867-LexA*) and *Tm9* neurons (*SS00307-spGal4*) (top), anti-LOVIT labels R8 axons in the medulla (middle), and overlap image (bottom); **right**, an expanded view of the white boxes in left panels. **g**, Ectopic expression of ort in AMA neurons. **Left**, representative histamine-induced responses in AMA neurons of wild-type flies (top) and transgenic flies with ort expressed ectopically in AMA neurons; **right**, pooled data of peak response amplitudes (n = 6). Histamine: 1 mM, duration of 250 ms. Pooled data are mean ± s.e.m. *P < 0.05; **P < 0.01; ***P < 0.001. Statistical analysis is summarized in Supplementary Table 2.

In contrast to the conventional view that light hyperpolarizes the second-order visual neurons^24^, our findings showed that AMA neurons depolarized following light stimulation. This depolarization was lost when both Rh5 and Rh6 were mutated, and restored with *norpA* rescued in R8 photoreceptors of *norpA*^*P41*^ mutant flies (Fig. 3d,e and Extended Data Fig. 6a). Furthermore, the nicotinic ACh receptor antagonist mecamylamine (MCA)^12^, but not the histamine receptor blocker cimetidine (CIM), blocked light-induced depolarization of AMA neurons (Fig. 3e). Finally, the depolarizing response was completely lost following conditional knockout of either *ChAT* or *VAChT* in R8 photoreceptors, but remained unchanged when HDC or histamine receptors were mutated (Fig. 3e). We also observed conventional light-induced hyperpolarization of postsynaptic neurons of R8 photoreceptors, including L1, Tm9 and Tm20 (Fig. 3f and Extended Data Fig. 6b). Exogenous histamine directly induced CIM-sensitive hyperpolarization in L1, Tm9 and Tm20 (Extended Data Fig. 6c), and genetic or pharmacological disruption of histamine receptors abolished R8-mediated hyperpolarization (Fig. 3f and Extended Data Fig. 6b). A small MCA-sensitive depolarization was apparent in the absence of histamine signalling (Fig. 3f and Extended Data Fig. 6b), indicating that even though R8 photoreceptors drive a minor excitatory response, they predominantly drive hyperpolarization of L1, Tm9 and Tm20 through histamine signalling. Together, these results demonstrate that physiological activation of R8 photoreceptors depolarizes AMA neurons through ACh signalling but hyperpolarizes L1, Tm9, and Tm20 through histamine signalling. The histamine transmission from R8 photoreceptors to L1 may account for the observed motion detection by R8 photoreceptors (Fig. 2e) as L1 is one of the two major motion pathways^48^.

We next investigated whether R8 photoreceptors release ACh and histamine at the same or different presynaptic sites. We developed a modified GRASP method by separately targeting postsynaptic membrane of two neurons with two complementary split-GFP fragments that are fused to intercellular adhesion molecule 5 (ICAM5), so that reconstituted GFP signals would occur only when the two postsynaptic neurons share the same presynaptic terminal in a polyadic synapse (Extended Data Fig. 6d,e). We termed this method polyadic synaptic GRASP. Strong polyadic synaptic GRASP signals were observed in M1–M3 between AMA and Tm9 or Tm20 neurons (Fig. 3g and Extended Data Fig. 6f), implying that these neurons can possibly encounter both ACh and histamine in the same polyadic R8 synapse, which is also implicated in another study^26^. To verify this, we ectopically expressed *ort* in AMA neurons of triple-mutant *CHO* flies (Extended Data Fig. 6g). Unlike the monophasic depolarization in wild-type flies, biphasic responses were observed in *ort*-expressing AMA neurons following light stimulation (Fig. 3h). Furthermore, a depolarizing component mediated by ACh was revealed in the presence of CIM and a hyperpolarizing component mediated by histamine was revealed in the presence of MCA (Fig. 3h). Thus, AMA neurons encounter both ACh and histamine, whereas the monophasic responses in wild-type flies are due to ACh but not histamine.

On the basis of these data, we propose a model in which histamine and ACh cotransmission is segregated postsynaptically (Fig. 3i). Following light stimulation, R8 photoreceptors depolarize and release both histamine and ACh at the same axonal terminals, but postsynaptic neurons expressing distinct transmitter receptors segregate these signals. Consequently, AMA neurons are depolarized whereas L1 and Tm neurons are hyperpolarized by light.

## A three-node circuit encodes irradiance

**Fig. 4:**
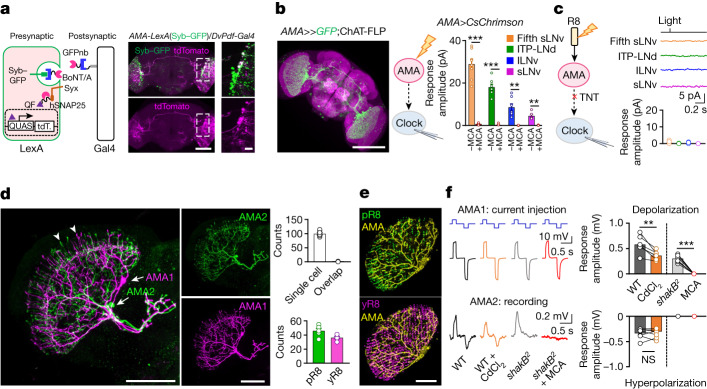
A three-node circuit for circadian photoentrainment. **a**, AMA neurons are presynaptic to clock neurons. Left: schematic of retrograde BAcTrace tracing; right: AMA neurons overlap with the BAcTrace-labelled presynaptic neurons of clock neurons. Scale bars, 20 µm. **b**, AMA neurons excite clock neurons through ACh signalling. Left: GFP expression intersected by *ChAT-FLP* and *AMA-Gal4*; scale bar, 100 µm. Middle: schematic of simultaneous optogenetic activation and patch-clamp recordings. Right: pooled responses of clock neurons to optogenetic activation of AMA neurons (with or without MCA). Light: 617 nm, 2 ms, 2.22 × 10^9^ photons μm^−2^ s^−1^. **c**, R8 photoreceptors excite clock neurons through AMA neurons. Left: schematic of TNT blockade of AMA transmission; right: a complete loss of R8-mediated light responses in clock neurons of *AMA-Gal4* > *TNT* flies (top) and pooled data (bottom). Light: 470 nm, 2 ms, 5.56 × 10^7^ photons μm^−2^ s^−1^. **d**, Spatial irradiance integration by AMA neurons. Left: arborization of two MCFO-labelled AMA neurons; arrows indicate cell bodies and arrowheads indicate multicolumnar arborization. Middle: single MCFO-labelled AMA neurons. Top right: pooled counting data for dendritic branches of single AMA neurons and for dendritic branch overlaps between two AMA neurons. Scale bars, 50 µm. **e**, Co-labelling of pR8 or yR8 photoreceptors and AMA neurons. Pooled data counting the overlap between AMA dendritic branches and photoreceptor axons are shown on the bottom left. Scale bar, 50 µm. **f**, Electrical and chemical synapses among AMA neurons. Left: representative dual recordings (with current injection to AMA1) in wild-type flies (with or without 50 µM MCA) or *shakB*^*2*^ mutant flies (with or without 100 µM CdCl_2_); right: pooled peak response amplitudes of the AMA2 neuron. Hyperpolarization current: −30 pA; depolarization current: 30 pA. Pooled data are shown as mean ± s.e.m. **P < 0.01; ***P < 0.001; NS, not significant. Statistical analysis is summarized in Supplementary Table 2.

**Extended Data Fig. 7:**
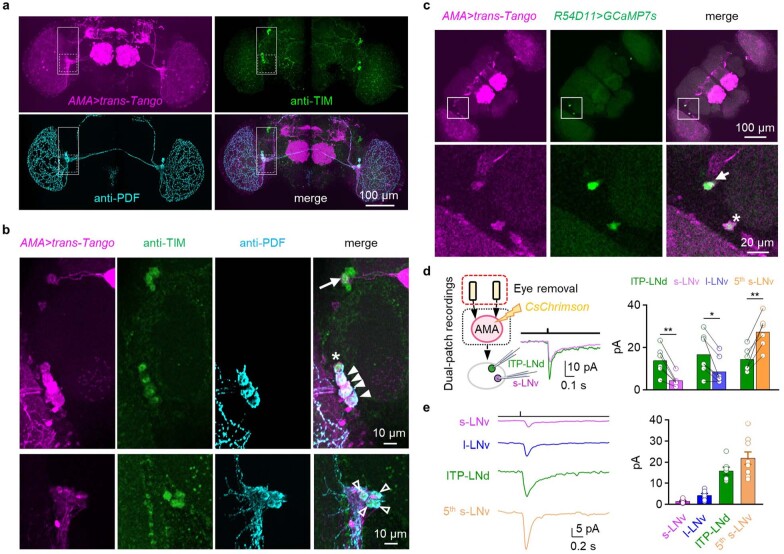
Clock neurons are postsynaptic to AMA neurons. **a**, Clock neurons overlap with postsynaptic neurons of AMA neurons. Double-labeling clock neurons (with anti-TIM and anti-PDF) shows overlap with tdTomato-labeled postsynaptic neurons of AMA neurons (*VT037867-Gal4*>*trans-Tango*). **b**, An expanded view of the white rectangles (top) and the dashed boxes (bottom) in **a**. Arrow: LNds; asterisk: 5^th^ s-LNv; arrowhead: l-LNv; unfilled arrowhead: s-LNv. **c**, Evening clock neurons are postsynaptic to AMA neurons. **Top**, Co-labeling ITP-LNd and 5^th^ s-LNv (*R54D11-LexA* > *GCaMP7s*) and postsynaptic neurons of AMA neurons (*VT037867-Gal4>trans-Tango*); **bottom**, an expanded view of the white boxes in top panels. Arrow: LNds; asterisk: 5^th^ s-LNv. **d**, Varied synaptic strengths between AMA neurons and clock neurons. **Left**, schematic of dual-patch recordings; **middle**, representative responses of a pair of ITP-LNd and s-LNv to optogenetic activation of AMA neurons; **right**, pooled peak responses (lines represent pairs of neurons simultaneously recorded with dual patch-clamp recordings). Light: 617 nm, 2 ms, 2.22 × 10^9^ photons/μm^2^/s. **e**, R8s differentially excite different clock neurons. **Left**, representative R8-driven light responses of different clock neurons; **right**, pooled peak response amplitudes (mean ± s.e.m.). Light: 470 nm, 2 ms, 5.65 × 10^7^ photons/μm^2^/s. Pooled data are mean ± s.e.m. *P < 0.05; **P < 0.01. Statistical analysis is summarized in Supplementary Table 2.

To investigate how AMA neurons relay non-histaminergic signals to the circadian clock, we immunostained the clock protein Timeless (Tim) and pigment dispersing factor (Pdf) to examine whether anterograde *trans*-Tango tracing can label clock neurons as their postsynaptic neurons, and found that clock neurons, including sLNv, lLNv, fifth sLNv and ITP-LNd, are postsynaptic to AMA neurons (Extended Data Fig. 7a–c). Moreover, retrograde trans-synaptic tracing using botulinum-activated tracer (BAcTrace)^49^ confirmed that AMA neurons are presynaptic to clock neurons (Fig. 4a). We verified their functional connections using optogenetic activation of AMA neurons, which induced excitatory postsynaptic currents with increasing amplitudes from sLNvs, to lLNvs, ITP-LNd and the fifth sLNv (Extended Data Fig. 7d). Thus, AMA neurons make stronger connections with evening cells (fifth sLNv and ITP-LNd) than with arousal (lLNvs) and morning cells (sLNvs). Indeed, R8-driven light responses in clock neurons also showed the same order of strength (Extended Data Fig. 7e). MCA abolished AMA-driven excitatory postsynaptic currents in clock neurons and genetic intersection confirmed that AMA neurons are acetylcholinergic (Fig. 4b). Moreover, when transmission from AMA neurons was blocked, light activation of R8 photoreceptors failed to excite clock neurons (Fig. 4c). Together, these data indicate that R8 photoreceptors, AMA and clock neurons form a three-node circuit that drives circadian photoentrainment to low-light-intensity LD cycles, and that they do so by means of cholinergic transmission. However, more experiments are needed to prove that AMA neurons directly drive photoentrainment, which relies on the development of new tools to manipulate this circuit in the future.

**Extended Data Fig. 8:**
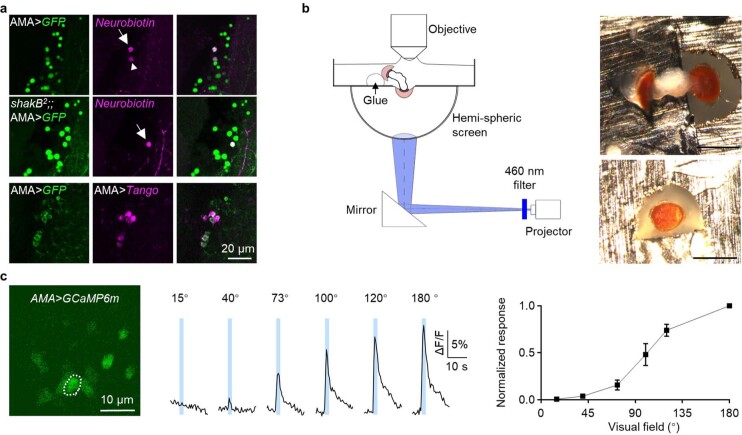
Mutual synapse and receptive field of AMA neurons. **a**, Mutual electrical/chemical synapses between AMA neurons. **Top**, neurobiotin injection to an AMA neuron labels another AMA neuron; **middle**, loss of neurobiotin coupling in AMA neurons of *shakB*^*2*^ flies; **bottom**, AMA neurons are their own postsynaptic neurons labeled by *trans*-Tango. Arrows: neurobiotin-injected cell; arrowhead: neurobiotin-coupled cells. **b**, Mapping receptive field of AMA neurons. **Left**, Schematic of preparations for mapping receptive field. The recording chamber was made of a thin sheet of stainless steel (20 μm thick), with a hole slightly larger than a compound eye. Fly brains with intact eyes were dissected, and one compound eye was exposed to the air of the bottom of the chamber. The other compound eye was stabilized in the chamber with glue. A hemispherical screen (diameter of 40 mm) was placed under the chamber, with the exposed compound eye facing the center of the screen. Light spots were projected to the screen from a back projector via a reflecting mirror. Light simulation of 460 nm was used to avoid interference with the fluorescence-imaging detector. Calcium responses reported by GCaMP6m were acquired with a two-photon microscopy and fluorescence excitation by a two-photon laser of 920 nm. **Right**, a representative top view of the brain preparation (top) and bottom view of the exposed compound eye (bottom). Scale bar: 500 µm. **c**, Large receptive field of AMA neurons. **Left**, representative calcium imaging of AMA neurons; **middle**, representative response traces of AMA neurons to visual stimuli; **right**, pooled normalized peak responses against spot size. Light stimulation: 460 nm, 2.5 s, 2.36 × 10^6^ photons/μm^2^/s, spot size: 15°, 40°, 73°, 100°, 120°, and 180°. Blue bars show the timing of light stimulation. Pooled data are mean ± s.e.m. Statistical analysis is summarized in Supplementary Table 2.

As each AMA neuron innervates many visual columns in the medulla, we examined how it integrates the spatial and spectral irradiance information. A single MCFO-labelled AMA neuron showed widespread arborization in about 100 visual columns (Fig. 4d) and innervation of both blue-sensitive pR8 and green-sensitive yR8 columns (Fig. 4e). Little overlap in columnar arborization was found between two random AMA neurons (Fig. 4d). On the other hand, neurobiotin injection revealed dye coupling among AMA neurons, which was absent in *shakB*^*2*^ mutant flies (Extended Data Fig. 8a). This implies that innexin 8 mediates electrical coupling between AMA neurons, which we verified by dual patch-clamp recordings (Fig. 4f). We also found that AMA neurons were mutually coupled via cholinergic chemical synapses: *trans*-Tango tracing revealed AMA neurons as their own postsynaptic neurons (Extended Data Fig. 8a) and mutual excitation between AMA neurons was reduced by a non-selective chemical transmission blocker cadmium (Fig. 4f). Furthermore, we found that light-triggered calcium responses of a single AMA neuron increased with the size of stimulating light spots and reached maximum only when the eye was fully covered by light stimulation (Extended Data Fig. 8b,c), implying irradiance integration through its medulla-wide dendritic arborization. Thus, AMA neurons can integrate spatial and spectral irradiance signals through their multicolumnar and dual pR8 and yR8 columnar arborization, respectively, and also share irradiance signals through their mutual electrical and chemical coupling.

## Histamine feedback supports ACh release

**Fig. 5:**
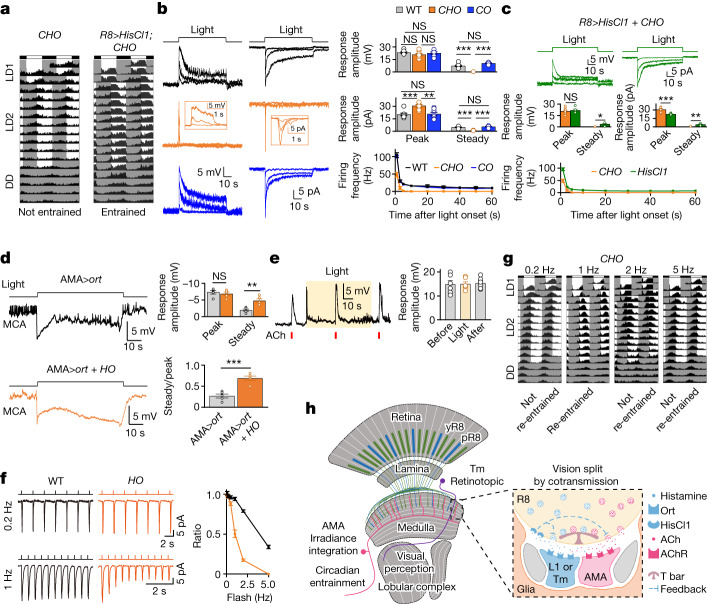
Histamine feedback sustains ACh cotransmission. **a**, ACh-mediated circadian photoentrainment requires HisCl1. Average actogram of *CHO* flies (left: *n* = 61) and *CHO* flies with HisCl1 rescued in R8 photoreceptors (right: *n* = 71). **b**, Light step-induced AMA responses in wild-type (black), *CHO* (orange) and *CO* (blue) flies. Left: representative voltage responses (current clamp). Middle: representative current responses (voltage clamp). Right: pooled peak and steady voltage response amplitudes (top), current response amplitudes (middle) and firing rates (bottom). Light: 470 nm, 60 s, intensities of 0.0094, 0.19 and 1.88 × 10^6^ photons μm^−2^ s^−1^. **c**, HisCl1 in R8 photoreceptors restored steady depolarization in *CHO* flies. Top: representative recordings in *CHO* flies with HisCl1 rescued in R8 photoreceptors; middle and bottom: pooled data. Light: 470 nm, 60 s, intensities of 0.0094, 0.19 and 1.88 × 10^6^ photons μm^−2^ s^−1^. **d**, Negative histamine feedback to histamine release. Left: Histamine-mediated hyperpolarization in *ort*-expressing AMA neurons is increased in *HO* flies when ACh-mediated depolarization is blocked by MCA. Right: pooled steady and peak response amplitudes and their corresponding ratios. Light: 470 nm, 60 s, 1.88 × 10^6^ photons μm^−2^ s^−1^. **e**, Normal ACh sensitivity in *CHO* flies. Left: representative ACh-induced depolarization before, during and after light stimulation; right: pooled data. Light: 470 nm, 60 s, 1.88 × 10^6^ photons μm^−2^ s^−1^. **f**, Frequency dependence of light-pulse-induced responses in AMA neurons. Left: representative responses to light pulses of 0.2 Hz and 1 Hz; right: pooled peak response ratios between the first and fiftieth pulses. Light pulses: 470 nm, duration of 100 ms, 5.65 × 10^7^ photons μm^−2^ s^−1^. **g**, Circadian photoentrainment of *CHO* flies to brief light pulses. Average actograms to 100-ms flashes at 0.2 Hz (*n* = 63), 1 Hz (*n* = 65), 2 Hz (*n* = 60) and 5 Hz (*n* = 54). **h**, A model of negative histaminergic feedback in R8 photoreceptors. Pooled data are shown as mean ± s.e.m. *P < 0.05; **P < 0.01; ***P < 0.001; NS, not significant. Statistical analysis is summarized in Supplementary Table 2.

**Extended Data Fig. 9:**
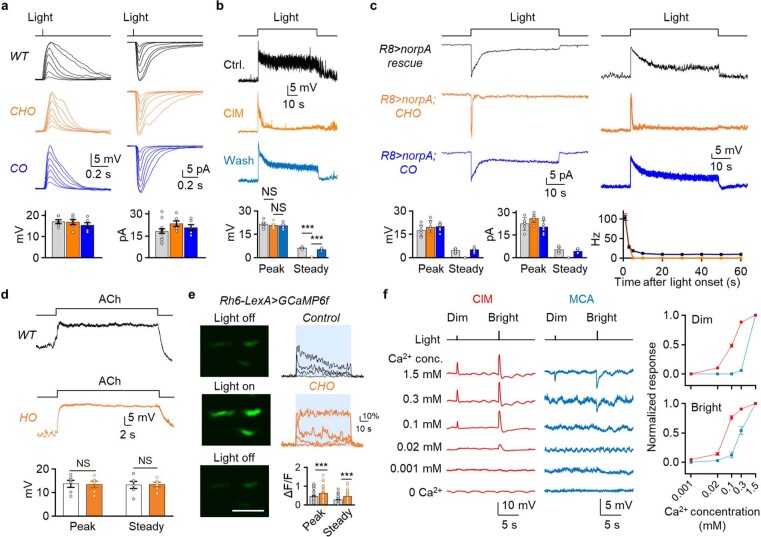
ACh release sustained by histamine feedback in R8s. **a**, Intact light-pulse-triggered responses in AMA neurons of CHO flies. **Top**, representative voltage responses (left) and representative current responses (right). Light stimulation: 470 nm, 2 ms, intensities of 0.51, 0.94, 1.51, 1.88, 2.82, 4.52, and 6.59 × 10^7^ photons/μm^2^/s. **Bottom**, pooled data to light of 470 nm, 2 ms, and 5.65 × 10^7^ photons/μm^2^/s. **b**, CIM reduced steady depolarization of AMA neurons. **Top**, representative recordings in normal saline, CIM, and after washout of CIM; **bottom**, pooled peak amplitudes (left) and steady response amplitudes (right). Light: 470 nm, 60 s, 1.88×10^6^ photons/μm^2^/s. **c**, **Top**, representative light step-triggered steady currents (left), depolarization and spike firing (right); **bottom**, pooled peak and steady membrane depolarization (left), inward currents (middle), and firing rates (right). Light stimuli: 470 nm, 60 s, 1.88×10^6^ photons/μm^2^/s. **d**, Normal AChR functions in AMA neurons of *HO* flies. Representative response to 1 mM ACh in wild-type (top) and *HO* flies (middle), and pooled data (bottom). Note, there is a hyperpolarization after washing out ACh (top). **e**, Enhanced light-triggered calcium influx in R8 axon terminals of *CHO* flies. **Left**, representative calcium responses; **right**, representative calcium response families of yR8 axons in wild-type (top) and *CHO* flies (middle), and pooled peak and steady response amplitudes (bottom). Light: 470 nm, 60 s, intensities of 0.33, 1.81, 4.22, and 9.64 × 10^5^ photons/μm^2^/s. Blue bars indicate timing of light stimulation. Scale bar: 10 µm. Pooled data are mean ± s.e.m. ***P < 0.001. **f**, Distinct calcium sensitivity of histamine and ACh release. **Left**, representative flash-triggered responses of ort-expressing AMA neurons in the presence of CIM (left) or MCA (right) at different calcium concentrations; **right**, pooled data. Light stimulation: 470 nm, 2 ms, 9.41 × 10^6^ photons/μm^2^/s (dim) and 6.78 × 10^7^ photons/μm^2^/s (bright). Pooled data are mean ± s.e.m. **P < 0.01; ***P < 0.001. Statistical analysis is summarized in Supplementary Table 2.

Although double-mutant flies (*CO*; *cry*^*02*^*,ort*^*1*^) can re-entrain to new LD cycles (Fig. 2h), *CHO* flies that additionally lack the histamine receptor HisCl1 were unable to photoentrain and HisCl1 rescue in R8 photoreceptors restored photoentrainment (Fig. 5a), consistent with a previous report^9^. Thus, HisCl1 seems to be indispensable for ACh-mediated circadian photoentrainment through an unknown mechanism. We investigated this mechanism by comparing light-induced responses of AMA neurons in the *CO* and *CHO* flies. Brief pulses of light elicited similar responses (Extended Data Fig. 9a), but long steps of light elicited an initial transient depolarization followed by a relaxed steady depolarization in *CO* flies and just an initial transient in *CHO* flies (Fig. 5b). As HisCl1 rescue in R8 photoreceptors restores circadian photoentrainment^9^ (Fig. 5a), we tested the same rescue on light-induced responses of AMA neurons and observed restoration of the steady depolarization in *CHO* flies (Fig. 5c). Moreover, pharmacological blockade of histamine receptors in wild-type flies also abolished the steady depolarization (Extended Data Fig. 9b), and the histaminergic feedback in R8 photoreceptors sufficed to sustain steady depolarization in the transgenic flies with only R8 but not R7 photoreceptors capable of light detection (with *norpA* rescue only in R8 photoreceptors; Extended Data Fig. 9c). By contrast, in the absence of HisCl1, we observed an increase of light-triggered steady hyperpolarization in *ort*-expressing AMA neurons in the presence of MCA (Fig. 5d), implying an increase of continuous histamine release. Together, these results revealed that the cell-autonomous histaminergic feedback in R8 photoreceptors is required for continuous ACh release but not for continuous histamine release during long light illumination.

Such feedback could maintain continuous presynaptic ACh release or prevent desensitization of postsynaptic ACh receptors. However, AMA neurons in *CHO* flies were able to respond to exogenous application of pulses of ACh in the same way before, during and after a long step of light (Fig. 5e) and were able to react with prolonged depolarizing responses to steps in ACh (Extended Data Fig. 9d), suggesting that postsynaptic ACh receptors are not affected by this feedback. We next investigated whether histaminergic feedback affects presynaptic transmitter release. Calcium imaging in R8 axons with GCaMP6 showed that steps of light induced a slight increase of calcium influx in the absence of HisCl1 (Extended Data Fig. 9e). Together with the evidence that ACh release from R8 photoreceptors showed a higher sensitivity to calcium changes than histamine (Extended Data Fig. 9f), the loss of ACh-mediated steady responses may reflect no continuous ACh release possibly owing to local depletion in the absence of HisCl1-mediated negative feedback.

**Extended Data Fig. 10:**
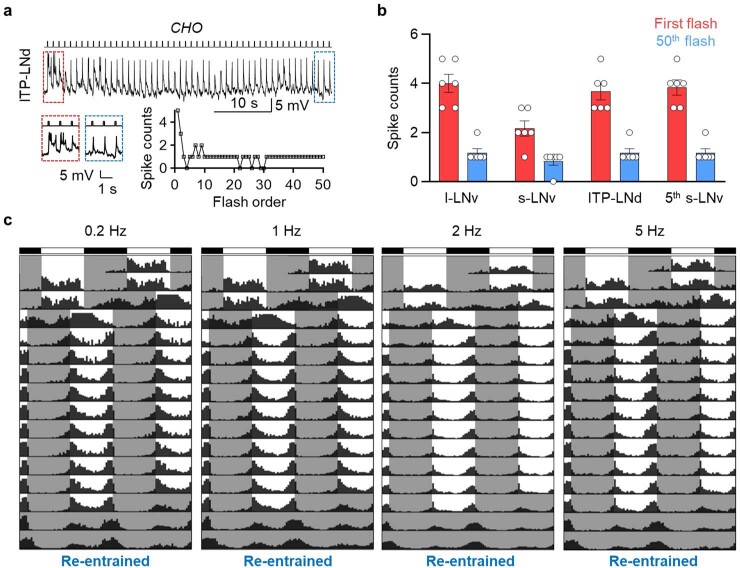
Flash frequency-dependent photoentrainment. **a**, **Top**, representative response to 1 Hz flashes of ITP-LNd in *CHO* flies; **bottom**, an expanded view of the first three (red box) and last three (blue box) responses (left), and spike counts (right). **b**, Pooled data of the first and the 50^th^ flash-induced spikes in different clock neurons of *CHO* flies. Light: 470 nm, 100 ms, 4.71 × 10^7^ photons/μm^2^/s. Pooled data are mean ± s.e.m. **c**, Average actograms to light flashes (duration of 100 ms) of wild-type flies at 0.2 Hz (n = 15), 1 Hz (n = 20), 2 (n = 23), and 5 Hz (n = 21). LD1: 200 lux (white light) together with 25 °C/18 °C temperature cycles; LD2: 0.5 lux (white light) at 25 °C; DD: 25 °C. Statistical analysis is summarized in Supplementary Table 2.

Given that AMA neurons can respond to short pulses of light in the absence of HisCl1 (Extended Data Fig. 9a), we wondered whether they can maintain their responses to long trains of brief pulses of light. For a given intensity and pulse duration, AMA neurons sustained responses to trains of low-frequency pulses (≤1 Hz) but not high-frequency pulses (Fig. 5f). Moreover, clock neurons responded to short pulses of light with a similar frequency dependence in *CHO* flies (Extended Data Fig. 10a,b). Notably, *CHO* flies exhibited circadian photoentrainment when the light phase of LD cycles comprised brief light pulses at 1 Hz but not at other tested frequencies including the 0.2 Hz that triggered robust sustained responses every 5 s (Fig. 5g), indicating that the circadian clock can be entrained only through proper temporal integration of clock-resetting molecules produced by light-pulse-triggered transient electrical responses. By contrast, wild-type flies entrained to all the tested frequencies (Extended Data Fig. 10c). Together, these data indicate that the dual transmitter signalling in R8 photoreceptors interacts through a negative histaminergic feedback (Fig. 5h).

## Discussion

We have shown that R8 photoreceptors split visual perception and circadian photoentrainment by co-transmitting two neurotransmitters. This visual segregation is further supported by postsynaptic circuitry in the medulla, such that each unicolumnar neuron mainly receives histaminergic inputs from a single R8 photoreceptor (thus transmitting the fine retinotopic signal), whereas each multicolumnar AMA neuron integrates cholinergic inputs from up to 100 R8 photoreceptor cells (thus integrating the irradiance visual signal). These AMA neurons directly excite downstream clock neurons, forming a shallow three-node circuit for circadian photoentrainment. Thus, this clock-entrainment circuit integrates irradiance signals directly from conventional photoreceptors, bypassing the downstream image-forming processing circuit to avoid a less efficient reconstruction of irradiance signals from the already highly processed signals in the contrast-encoding visual pathways. Notably, the AMA neurons overlap with our recently discovered xCEOs that sustain circadian timekeeping of free-running circadian clocks^44^. Together, our findings reveal that the AMA neurons and xCEOs play crucial roles in circadian timekeeping, resetting circadian clocks to synchronize their endogenous rhythms to local time by integrating irradiance signals from retinal photoreceptors under LD cycles and sustaining free-running circadian timekeeping through their intrinsic rhythmic electrical oscillations under constant darkness.

Our observation that the compound eye-driven electrical responses in clock neurons were reduced by half in the absence of histamine receptors (Fig. 1b) suggests that conventional photoreceptors other than R8 photoreceptors (for example, R1–R6) can use histamine-mediated circuitry pathways to excite clock neurons for circadian photoentrainment^10,12,15^. The downstream circuits of R1–R6 photoreceptors might reconstruct irradiance signals from image-forming signals via yet-to-be-uncovered mechanisms as mammalian rod and cone pathways do^18–20^, indicating the mechanisms underlying irradiance encoding for circadian photoentrainment by conventional photoreceptor pathways might have evolved convergently.

We also identified an unexpected crosstalk between the image-forming vision and circadian photoentrainment. The chloride channel HisCl1 mediates negative feedback of histamine in R8 photoreceptor cells, thus dynamically reducing photoreceptor depolarization during long light stimulation. This feedback regulation tunes ACh release to avoid its local depletion so that the irradiance signal can be continuously transmitted from R8 photoreceptors to clock neurons during the entire light phase.

Our work demonstrates that visual perception and circadian photoentrainment can be segregated as early as the first-order synapses in the visual system, providing a simple yet robust mechanism to enact distinct sensory functions. Furthermore, although cotransmission or co-release of neurotransmitters is an emerging principle in brain research^50^, its behavioural significance remains largely unknown. Our finding that cotransmission from the same photoreceptor cells enables segregation and translation of distinct visual features into different behaviours also paves the way for understanding this key, indispensable aspect of the nervous system.

## Methods

### Flies

Flies were reared on standard cornmeal medium under a 12-h/12-h LD cycle at a humidity of 60% and temperature of 25 °C, except for *trans*-Tango tracing flies, which were raised at 18 °C. At 1–2 days after eclosion, flies were used for patch-clamp recordings regardless of sex. Recordings from *Rh6-hid*,*rpr* flies were carried out on the third day after eclosion.

The details of fly sources are listed in Supplementary Table 3, and fly genotypes are listed in Supplementary Table 1.

### Generation of transgenic flies

To generate the *Rh6-hid*,*rpr* construct, a 2.9-kilobase enhancer was amplified from *Rh6–eGFP* flies (BDSC7461) with HindIII (5′) and NotI (3′) sites added to the primers. Primer sequences were as follows—forward: 5′-GCACCCGTGGCCAGGGCCGCAAGCTTATGAACATGTTGCCTCATTGAATC-3′; reverse: 5′-TCGATCCCCGGGCGAGCTC GGCGCGCCCACACCCATTTTGCTCAGTGCATCT-3′. The *head involution defective-2A-reaper* (*hid,rpr*) sequence was amplified from *UAS-hid*,*rpr* with NotI (5′) and XbaI (3′) sites added to the primers. Primer sequences were as follows—forward: 5′-ACCAGTACCAGCCATTCGAAGCGGCCGCGCCACCATGGCCGTGCCCTTT-3′; reverse: 5′-GTTATTTTAAAAACGATTCATTCTAGATCATTGCGATGGCTTGCGATA-3′. The *Rh6-hid,rpr* fragment was then inserted into the *pUASTattB* vector, and the *p10* sequence was inserted downstream of the *hid,rpr* sequence. To generate transgenic flies, the above constructs were injected and integrated into the *attp5* landing sites through phiC31-mediated gene integration.

To generate the *ort-QS* construct, the *ort* promoter was amplified from the *ort*^*C1-3*^*-Gal4* fly^38^ with HindIII (5′) and NotI (3′) sites added to the primers. Primer sequences were as follows—forward: 5′-ACCCGTGGCCAGGGCCGCAAGCTTGCCAAACACAAGTAAAAAGTTTGC-3′; reverse: 5′-GGGATGGTGTTCATTTTGCGGCCGCTTTAATGTGAGCTCTTTCTGTGTGG-3′. The promoter fragment was then inserted into the *pUASTattB* vector, and the *QS-P10* sequence was inserted downstream of the *ort* promoter. To generate transgenic flies, the above constructs were injected and integrated into the *VK00005* landing sites through phiC31-mediated gene integration.

To generate the *LexAop2-post-GFP*^*11*^ construct, the mouse neuroligin 1 (*Nlgn1*) fragment sequence was amplified from the *UAS-post-GFP*^*1–10*^ fly, with NotI (5′) sites added to the primers. Primer sequences were as follows—forward: 5′-CTTATCCTTTACTTCAGGCGGCCGCAAAATGGCACTTCCTAGATGTATGTGGCCTA-3′; reverse: 5′-CACATATTCGTGCAGCACCATGTGGTCGCGGGAAGCGTCATCCAGCTTCTGTGAAAG-3′. *Telencephalin* (*TLN*) and the linker sequence was amplified from the *UAS-post-GFP*^*1–10*^ fly, with XbaI (3′) sites added to the primers: Primer sequences were as follows—forward: 5′-GGTGCTGCACGAATATGTGAACGCTGCTGGCATCACAGGGACGGGCGGATCTGGCGGATCT-3′; reverse: 5′-GTTATTTTAAAAACGATTCATTCTAGATCAGGAAGATGTCAGCTGGATA-3′. The *Nlgn1-GFP*^*11*^*-TLN* sequence was then amplified using the above two sequences as templates. Primer sequences were as follows—forward: 5′-CTTATCCTTTACTTCAGGCGGCCGCAAAATGGCACTTCCTAGATGTATGTGGCCTA-3′; reverse: 5′-GTTATTTTAAAAACGATTCATTCTAGATCAGGAAGATGTCAGCTGGATA-3′.

The above sequence was then inserted into the *LexAop2-IVS-p10* vector (constructed from Addgene, 36431). To generate transgenic flies, the above constructs were then injected and integrated into the *VK00005* landing sites through phiC31-mediated gene integration.

The *UAS-ChAT-sgRNA* and *UAS-VAChT-sgRNA* constructs were designed by inserting single guide RNAs (sgRNAs) into the pMt:sgRNA^3×^ vectors based on pACU2, with rice transfer RNA used to separate different sgRNAs.

### Electrophysiological recordings

Fly dissections, preparation visualization and patch-clamp recordings were carried out as described previously^12,44^.

AMA neurons and clock neurons exhibit robust rhythmic bursting activity^44^. To reduce its interference with the analysis of light-induced responses, we selected the voltage recordings with weak rhythmic bursting activity or averaged more than 15 traces for the current recordings by flattening baselines. Data were processed and visualized with Clampfit v10 (https://www.moleculardevices.com/), Matlab R2020b (https://www.mathworks.com/products/matlab.html), Origin 2018 (https://www.originlab.com/) and GraphPad Prism 9 (https://www.graphpad.com/scientific-software/prism/).

As for the recordings on AMA neurons, we selected the ten pairs of VT037867-labelled neurons in the accessory medulla region for patch-clamp recordings as they have the same morphological features as AMA neurons, on the basis of their single-cell morphology as revealed by MCFO labelling (Extended Data Fig. 4c). Furthermore, we confirmed that this group of neurons are indeed the AMA neurons on the basis of their single-cell morphology that is revealed by neurobiotin injection^12^ through the patch-recording electrode after whole-cell recordings (Extended Data Fig. 4d).

### Dual patch-clamp recordings

Dual patch-clamp recordings were carried out similarly to the single patch-clamp recordings above. The two target neurons were identified on the basis of GFP expression, and their cell bodies were exposed before patch-clamp recordings. After cell body exposure, two patch recording electrodes were sequentially positioned about 50 μm above the target neuron. The target neuron was then sequentially patched by the two electrodes one by one.

### Light stimulation

Light stimulation was carried out as described previously^12^. The light source was a 470-nm LED (M470L4, Thorlabs) controlled by an LED driver (LED D1B and DC 4100, Thorlabs) and connected to the fluorescence port of an A1 MP+ microscope through a liquid light guide. A light spot (diameter 400 μm) was projected onto the preparations, roughly covering the ipsilateral compound eye. Light intensity was calibrated with a power meter (model 1936-R, 918D-ST-UV, Newport) before and after the experiments. In both electrophysiological recordings and calcium imaging, light intensity was reported in the unit of photons μm^−2^ s^−1^. For example, for the monochromatic 470-nm light spot (with diameter of 400 µm), 3 mW is equivalent to 5.65 × 10^7^ photons μm^−2^ s^−1^. Brief or short light pulses were typically used in our electrophysiological recordings and calcium imaging. It was not possible to achieve an identical light condition in cellular recordings and behavioural studies.

### Drug delivery by fast-solution changes

A three-barrel tube (SF77B, Warner Instruments) was positioned about 200 μm from the brain preparation and controlled by a stepper (SF77B, Warner Instruments). External saline was perfused through the middle barrel to cover the brain preparation, and the drugs in the side barrels were switched to cover the preparations using a step motor, achieved within milliseconds. The timing and duration of drug application were controlled by Clampex and a Digidata 1440A. The following were used: 100 μM CdCl_2_ (20899, Fluka Sigma-Aldrich)^51^, 50 μM MCA (M9020, Sigma-Aldrich)^51^, 2 mM CIM (C4522, Sigma-Aldrich)^52^, 1 mM histamine (H7250, Sigma-Aldrich)^52^ and 1 mM ACh (A6625, Sigma-Aldrich)^53^. Note, Cd^2+^ is a nonspecific blocker of voltage-gated calcium channels, in addition to some voltage-gated potassium channels. Free calcium concentrations were calculated on the basis of the program provided by https://somapp.ucdmc.ucdavis.edu/pharmacology/bers/maxchelator/CaMgATPEGTA-NIST.htm.

### Optogenetic manipulation

CsChrimson was expressed in the target neurons with a specific Gal4 driver. All parental flies were raised on food containing 0.4 mM all-*trans*-retinal (ATR, R2500, Sigma-Aldrich), with progenies also fed 0.4 mM ATR-food for 3 days after eclosion. Fly vials were kept in darkness to avoid ATR degeneration and CsChrimson activation. Isolated brains (without compound eyes) were dissected under dim blue light, and a 625-nm LED (M625L4, Thorlabs) was used for optogenetic stimulation during patch-clamp recordings.

### In vivo two-photon calcium imaging

The chamber used for in vivo imaging was modified from a published version^54^. The bottom of the chamber was made of a stainless steel sheet (20-µm thick) with a rectangular hole (800 μm × 1,200 μm). Flies were anaesthetized on ice for 1 min, and then quickly inserted into the chamber hole with the dorsal brain facing upwards. Legs and thorax were stabilized with low-melting-point paraffin. The head was bent downwards to expose the posterior brain, keeping the compound eyes below the chamber. The chamber was filled with *Drosophila* saline, and a small window was opened in the dorsal brain to expose the axon terminals of the R8 photoreceptors.

Imaging was carried out with a Nikon A1 MP+ microscope equipped with a Maitai DeepSee Ti:sapphire ultrafast laser (Spectra-Physics). Excitation of GCaMP6f was achieved by a two-photon laser of 920 nm, and images (256 × 128-pixel resolution) were acquired with a DU4 PMT detector at 8 Hz. Full-field light stimulation (460 nm) was projected to the compound eyes through a condenser, whose duration and timing were controlled by a D1B LED driver through NIS-Element (https://www.microscope.healthcare.nikon.com/) and Clampex (Molecular Device). The relative change in fluorescence (Δ*F*/*F*) of manually selected regions of interest (ROIs) was analysed using NIS-Element.

### Mapping receptive field of AMA neurons

The recording chamber was similar to the in vivo imaging chamber except for a smaller chamber hole (500 μm × 500 μm), which is slightly larger than a compound eye. Fly brains with intact compound eyes were dissected, and one compound eye was inserted into the chamber hole and exposed to the air. The other compound eye was stabilized to the chamber with vacuum grease (Dow Corning). A hemispherical screen (diameter 40 mm) was placed under the chamber, with the exposed compound eye facing the centre of the screen. Light spots were projected to the screen from a back projector through a reflecting mirror. The duration and timing of visual stimuli were controlled by Matlab. Light stimulation of 460 nm was used to avoid interference with the fluorescence-imaging detector. Calcium responses reported by GCaMP6m were acquired with a Nikon A1 MP+ microscope equipped with a 25× water-immersion objective and fluorescence excitation by a two-photon laser of 920 nm.

### Neuronal tracing with photoactivatable GFP

*VT037867-Gal4,UAS-tdTomato/UAS-mSPA-GFP* was used for photoactivatable (PA) GFP^55^ labelling of the AMA neurons. Ex vivo brain preparations were stabilized in the chamber with the posterior brain facing upwards to expose the tdTomato-marked dorsal commissural track of AMA neurons. Photoactivation of PA-GFP was achieved with a Nikon A1 MP+ microscope and a two-photon laser of 720 nm. One cycle of photoactivation of the commissure track included 30 photoactivation pulses, each with a dwell time of 4.8 μs per pixel at an interval of 8 s. The cycle was repeated 10 times with a 30-s interval for a complete photoactivation episode. After a 10-min wait for GFP diffusion, the brain preparations were repositioned with the anterior side facing up, and images of the photoactivated cell bodies were acquired with a two-photon laser of 920 nm.

### Immunostaining

Flies were first dissected in phosphate-buffered saline (PBS) and fixed in 4% (w/v) paraformaldehyde (PFA, 157-8, Electron Microscopy Science) for 1 h on ice. Brains and compound eyes were then washed three times (15 min each wash) in PBST (0.5% v/v Triton X-100 in PBS). The fixed samples were incubated with penetration–blocking buffer (10% normal goat serum and 2% Triton X-100 in PBS) for 2 h at room temperature, followed by incubation with primary antibodies (rat anti-LOVIT, 1:100, a gift from T. Wang; rabbit anti-HA, 1:200, 3724, Cell Signaling Technology; mouse anti-nc82, 1:100, DSHB; mouse anti-Pdf, 1:4,000, Pdf C7, DSHB; mouse anti-V5 DyLight 549, 1:100, MCA2894D549GA, Bio-Rad; mouse anti-V5 DyLight 647, 1:10, MCA1360A647, Bio-Rad) at 4 °C for 24 h. After three washes in PBST, the samples were incubated with secondary antibodies (goat anti-rat Alexa Fluor 568, 1:200, ab175710, Abcam; goat anti-rabbit Alexa Fluor 488, 1:200, A27034, Thermo Fisher; goat anti-mouse Alexa Fluor 647, 1:200, A21235, Thermo Fisher; goat anti-mouse Alexa Fluor 568, 1:200, A11004, Thermo Fisher) at 4 °C overnight. The antibodies were diluted in antibody dilution buffer (1% normal goat serum and 0.25% Triton X-100 in PBS). The samples were washed three times before mounting in FocusClear (FC101, Cedarlane Labs).

### MCFO

For single- and two-cell labelling of AMA neurons, *VT037867-Gal4* flies were crossed with *MCFO-1* flies^56^. The flies were raised at 25 °C, and 1-day-old adult progenies were placed in empty vials and incubated in a 37 °C water bath for 3 min to induce flippase expression. On the third day after heat shock, the flies were fixed for immunostaining.

The fly brains were dissected in PBS and fixed in 4% PFA for 1 h on ice. Brains were then washed three times in PBST. The fixed brains were incubated with penetration–blocking buffer for 2 h at room temperature.

For single-cell labelling, brains were incubated with DyLight549-conjugated mouse anti-V5 (1:100, MCA2894D549GA, Bio-Rad) at 4 °C overnight. For co-labelling of AMA neurons with pR8 and yR8 photoreceptors, brains were incubated with DyLight647-conjugated mouse anti-V5 (1:10, MCA1360A647, Bio-Rad) at 4 °C overnight. For two-cell labelling, brains were first incubated with rabbit anti-HA primary antibody (1:100, 3724, Cell Signaling Technology) at 4 °C overnight. After three washes with PBST at room temperature, the brains were then incubated with goat anti-rabbit IgG-conjugated Alexa Fluor 488 (1:200, A27034, Thermo Fisher) and DyLight549-conjugated mouse anti-V5 (1:100) for 24 h. The brains were then washed three times in PBST and mounted in FocusClear.

### Chemoconnectomics intersection

We used an intersection strategy to identify the neurotransmitter used by R8 photoreceptors and AMA neurons. FLP knock-in flies (*ChAT-FLP*, *vGlut-FLP*, *Trh-FLP*, *TH-FLP*, *GAD1-LexA*, *LexAop-FLP*, *vGAT-LexA* and *LexAop-FLP*) were crossed with *Rh5-Gal4*, *Rh6-Gal4; UAS-FRT-Stop-FRT-GFP* flies. *ChAT-FLP* flies were crossed with *UAS-FRT-Stop-FRT-GFP; VT037867-Gal4* flies. The flies were raised at 25 °C and dissected within 2–3 days after eclosion. The fixed samples were washed three times with PBST, and then mounted in FocusClear without immunostaining.

### *trans*-Tango

We used *UAS-myrGFP*,*QUAS-mtdTomato(3×HA);trans-Tango* flies for anterograde tracing of R8 photoreceptors and AMA neurons. Progeny flies were maintained in 12-h/12-h LD cycles at 18 °C after eclosion and dissected at 10 or more days after eclosion. We used *ort-QS* for labelling the non-*ort* target of R8 photoreceptors. The brains were then fixed in 4% PFA for 1 h on ice. For direct observation of fluorescence, the samples were mounted in FocusClear after washing three times with PBST.

For co-labelling with clock neurons, whole flies were fixed at ZT20 with 4% PFA and 0.1% Triton X-100 in PBS for 2.5 h at room temperature. The flies were then washed with PBST and dissected in PBST. Subsequently, the brains were incubated with 10% normal goat serum and 0.1% Triton X in PBS for 4 h at room temperature, and then with rabbit anti-Tim (1:250) at 4 °C overnight, or together with mouse anti-Pdf (1:4,000, Pdf C7, DSHB). After washing three times with PBST (20 min each wash), the brains were incubated with goat anti-rabbit IgG conjugated with Alexa Fluor 488 (1:200, A27034, Thermo Fisher), or together with goat anti-mouse IgG conjugated with Alexa Fluor 647 (1: 200 A21235, Thermo Fisher) for 7 h at room temperature. Brains were then rinsed three times with PBST (20 min each wash) and mounted in FocusClear.

### BAcTrace

BAcTrace retrograde tracing was used to identify connections between clock neurons and AMA neurons. *VT037867-LexA*,*DvPdf-Gal4* flies were crossed with *LexAop2-Syb::GFP-P10 (VK37) LexAop-QF2::SNAP25::HIVNES::Syntaxin (VK18); UAS-B3Recombinase (attP2) UAS*<*B3Stop*<*BoNT/A (VK5) UAS*<*B3Stop*<*BoNT/A(VK27) QUAS-mtdTomato::HA* flies. The flies were raised at 25 °C, and progenies were dissected and fixed within 2-3 days after eclosion. After washing three times with PBST, the samples were mounted in FocusClear without immunostaining.

### GRASP

GRASP^57^ was used to examine the monosynaptic connections between photoreceptors and L1, Tm5, Tm9, Tm20, and AMA neurons. *LexAop-mCD4::spGFP*^*11*^ and *UAS-mCD4::spGFP*^*1–10*^ were driven by specific LexA and Gal4 drivers. The flies were dissected at 1 week after eclosion. Fly brains were dissected in PBS and fixed in 4% PFA for 1 h on ice. Brains were then washed three times in PBST. The fixed brains were incubated with penetration–blocking buffer for 2 h at room temperature. The brains were then incubated with mouse anti-nc82 primary antibody (1:100, DSHB) at 4 °C overnight. After three washes with PBST at room temperature, the brains were then incubated with goat anti-mouse IgG conjugated to Alexa Fluor 568 (1:200, A11004, Thermo Fisher) overnight. The brains were then washed three times in PBST, and mounted in FocusClear for imaging.

### Genetic and laser ablation of HB eyelets

The apoptosis genes *hid* and *rpr* were used to ablate HB eyelets. We first generated transgenic flies with *hid* and *rpr* expression using the Rh6 promotor. We found that *Rh6-hid*,*rpr* flies lost HB eyelets by the third day after eclosion. However, pR8 photoreceptors that also express Rh6 remained partially functional up to 2 weeks after eclosion. Therefore, our recordings from *Rh6-hid*,*rpr* flies were carried out on the third day after eclosion when HB eyelets were lost but most yR8 photoreceptors remained functional.

For laser ablation of HB eyelet axons, we followed our previous established method^12^.

### Removal of compound eyes and HB eyelets

Flies (1 day after eclosion) were dark-adapted over 30 min before the experiments. The fly head was dissected in pre-oxygenized *Drosophila* dissection saline. The compound eye and lamina were carefully removed under a dissection microscope (M125, Leica) with dim-red-light illumination, which also removed the HB eyelets as they are physically located between the compound eye retina and lamina.

### Image acquisition and processing

Images were acquired sequentially in 1-µm sections using the Nikon A1 MP+ microscope equipped with a 25× water-immersion objective (CFI75 Apochromat 25×C W, Nikon) at a resolution of 1,024 × 1,024 pixels. For co-labelling of single AMA neurons and pR8 and yR8 photoreceptors, and co-labelling of VT037867-Gal4-driven *trans*-Tango, anti-Tim and anti-Pdf, images were acquired in 1-μm sections on a Dragonfly spinning-disc confocal microscope equipped with 40× oil-immersion objective. Images were processed and rendered using NIS-Element (Nikon) and ImageJ (Fiji, https://fiji.sc/).

### Motion detection assay

We used flies within 2–4 days after eclosion for motion detection assays. After anaesthetization on ice for 20 s, the flies were tethered to a fine platinum wire with histoacryl (B. Braun Medical). Flies that recovered from anaesthesia within a few seconds were used for further experiments. The platinum wire was then fixed to a three-axis linear micromanipulator that facilitates the positioning of the fly on an air-supported plastic ball^54^. Using a calibration camera, each fly was positioned at the centre and 0.4 mm above the ball, allowing it to walk freely. Ball movement was tracked by two cameras with ADNS-6090 chips, with real-time data recorded using MATLAB and saved on a PC. Recordings were synchronized using TTL signals.

Visual stimuli were presented through a DMD projector (DLP4710EVM-LC, Texas Instruments) controlled by Psychtoolbox 3. The cylindrical screen covered the front 180° azimuth angle of the fly. The green and blue channels of the projector were used as visual stimuli, with a refresh rate of 120 Hz. For motion detection stimuli, the spatial wavelength was 30° and angular velocity was 180° s^−1^ or 60° s^−1^. Each trial lasted 5 s and randomly included clockwise and anticlockwise rotation stimuli. The stimulus was presented for 1 s. Trials were abandoned if the fly moved less than 50% of the total trial time, and the fly was removed if it failed in more than half of the trials. Each fly was tested for 30 min in total.

### Circadian photoentrainment assay

Locomotor activity was measured using the DAM2 system (TriKinetics). Individual male flies were placed in a glass tube at 25 °C and 60% humidity. Flies were first subjected to three 12-h/12-h LD cycles (200 lux, white light, equivalent to the intensity of 3.55 × 10^6^ photons µm^−2^ s^−1^ at 470 nm) combined with 25 °C/18 °C temperature cycles, and then subjected to 10 LD2 cycles (about 0.05 lux, white light, equivalent to the intensity of 2.29 × 10^3^ photons µm^−2^ s^−1^ at 470 nm) with 8-h phase delay at 25 °C, followed by constant darkness at 25 °C. A light intensity of 0.05 lux in LD2 cycles was used to study the NorpA-dependent photoentrainment by eye photoreceptors.

For pulse light photoentrainment, the flies were first subjected to three 12-h/12-h LD1 cycles (200 lux, white light) combined with 25 °C/18 °C temperature cycles, followed by nine LD2 cycles with 8-h phase delay at 25 °C (with the light phase composed of 0.2-, 1-, 2- or 5-Hz light pulses of 100-ms duration, 0.05 lux, white light), and then subjected to constant darkness at 25 °C. Light pulses were generated and controlled by microcontrollers (UNO, Arduino). Data were analysed and visualized with the ImageJ plugin Actogram J69.

### Statistical analysis

All experimental data are reported as mean ± s.e.m. The Shapiro–Wilk normality test was used to determine the normal distribution of samples. Comparisons between two groups were carried out using two-tailed paired or unpaired Student’s *t*-tests (normal distribution) or Mann–Whitney *U*-tests (non-normal distribution). Comparisons across multiple groups were assessed using one-way analysis of variance followed by Tukey’s post hoc test (non-normal distribution), or the Kruskal–Wallis test (non-normal distribution) followed by Dunn’s test. The statistical analysis is detailed in Supplementary Table 2.

### Reporting summary

Further information on research design is available in the Nature Portfolio Reporting Summary linked to this article.

## Online content

Any methods, additional references, Nature Portfolio reporting summaries, source data, extended data, supplementary information, acknowledgements, peer review information; details of author contributions and competing interests; and statements of data and code availability are available at 10.1038/s41586-023-06681-6.

### Supplementary information

Supplementary Information Supplementary Tables 1 (fly genotypes), 2 (statistical details) and 3 (fly strains used in this paper).

Reporting Summary

## Data Availability

Behavioural, electrophysiological and morphological raw data and additional information required to reanalyse the data reported in this paper are available from the corresponding author upon request.
