# Supplementary material for: A single photoreceptor splits perception and entrainment by cotransmission
Source: Nature. 2023 Oct 25;623(7987):562–70. doi: 10.1038/s41586-023-06681-6 (PMC10651484; doi:10.1038/s41586-023-06681-6)
Supplement: Supplementary file 1 — Supplementary Tables 1 (fly genotypes), 2 (statistical details) and 3 (fly strains used in this paper). [file 41586_2023_6681_MOESM1_ESM.pdf]

nature portfolio

https://doi.org/10.1038/s41586-023-06681-6

Supplementary information

A single photoreceptor splits perception and entrainment by cotransmission

In the format provided by the authors and unedited

A single photoreceptor splits perception and entrainment by cotransmission

Na Xiao[1,2,3,4,5,11], Shuang Xu[1,2,3,4,11], Ze-Kai Li[1,2,3,4,11], Min Tang[1,2,3,4,9], Renbo Mao[1,2], Tian Yang[1,2,3,4], Si-Xing Ma[1,2,3,4], Peng-Hao Wang[1,2,3,4], Meng-Tong Li[1,2,3,4,10], Ajay Sunilkumar[6], François Rouyer[6], Li-Hui Cao[7] & Dong-Gen Luo[1,2,3,4,5,8] 🖂

Affiliations:

[1]State Key Laboratory of Membrane Biology, School of Life Sciences, Peking University, Beijing, China.

[2]IDG/McGovern Institute for Brain Research, Peking University, Beijing, China.

[3]Peking-Tsinghua Center for Life Sciences, Academy for Advanced Interdisciplinary Studies, Peking University, Beijing, China.

[4]School of Life Sciences, Peking University, Beijing, China.

[5]Center for Quantitative Biology, Academy for Advanced Interdisciplinary Studies, Peking University, Beijing, China.

[6]Institut des Neurosciences Paris-Saclay, Universite Paris-Sud, Universite Paris-Saclay, CNRS, Gif-sur-Yvette, France.

[7]School of Basic Medical Sciences, Beijing Key Laboratory of Neural Regeneration and Repair, Capital Medical University, Beijing, China.

[8]Chinese Institute for Brain Research, Beijing, China.

[9]Present address: Department of Psychiatry, University of Texas Southwestern Medical Center, Dallas, TX, USA.

[10]Present address: Zuckerman Mind Brain and Behavior Institute, Columbia University, New York, NY, USA.

[11]These authors contributed equally: Na Xiao, Shuang Xu, Ze-Kai Li.

*Correspondence: dgluo@pku.edu.cn

Table of contents

Supplementary Table 1 | Fly genotypes……………………………………………………...3
Supplementary Table 2 | Statistical details ………………………………………………...10
Supplementary Table 3 | Fly strains used in this paper ………………………………20

Supplementary Tables

Supplementary Table 1 | Fly genotypes

Figures	Fly genotypes	Notes
Fig. 1a	*DvPdf-Gal4,UAS-GFP*	Labeling clock neurons (l-LNv, s-LNv, 5th s-LNv, ITP-LNd, and cry-negative LNd)
Fig. 1b	*DvPdf-Gal4,UAS-GFP*<br>*Hdc*^{JK910}*; DvPdf-LexA,LexAop-GFP*<br>*DvPdf-Gal4,UAS-GFP; HisCl1*^{134}*,ort*^{1}	Labeling clock neurons in WT, *Hdc*^{JK910}, or *HO* flies<br>*Hdc*^{JK910}: histamine synthetase mutant<br>*HO*: histamine receptors *HisCl1* and *ort* double mutants
Fig. 1c	*DvPdf-Gal4,UAS-GFP; HisCl1*^{134}*,ort*^{1}<br>*norpA*^{P41}*/y; DvPdf-Gal4,UAS-GFP; HisCl1*^{134}*,ort*^{1}	Labeling clock neurons in *HO*, or *norpA*^{P41} and *HO* flies<br>*norpA*^{P41}: mutant of PLC, which is essential for phototransduction in *Drosophila*
	DvPdf-Gal4,UAS-GFP/Rh6-hid,rpr; HisCl1^{134}*,ort*^{1}<br>*DvPdf-Gal4,UAS-GFP/Rh6-Gal4,UAS-GFP; HisCl1*^{134}*,ort*^{1}	Labeling clock neurons in *HO* flies (with H-B eyelets genetically ablated or laser-ablated)<br>hid and rpr: head involution defective and reaper, causing caspase-dependent apoptosis
Fig. 1d	*norpA*^{P41}*/y; UAS-norpA/DvPdf-LexA,LexAop-GFP; HisCl1*^{134}*,ort*^{1}<br>*norpA*^{P41}*/y; Rh1-Gal4,UAS-norpA/DvPdf-LexA,LexAop-GFP; HisCl1*^{134}*,ort*^{1}<br>*norpA*^{P41}*/y; Rh3-Gal4,UAS-norpA/DvPdf-LexA,LexAop-GFP; HisCl1*^{134}*,ort*^{1}<br>*norpA*^{P41}*/y; Rh4-Gal4,UAS-norpA/DvPdf-LexA,LexAop-GFP; HisCl1*^{134}*,ort*^{1}<br>*norpA*^{P41}*/y; Rh5-Gal4,UAS-norpA/DvPdf-LexA,LexAop-GFP; HisCl1*^{134}*,ort*^{1}<br>*norpA*^{P41}*/y; Rh6-Gal4,UAS-norpA/DvPdf-LexA,LexAop-GFP; HisCl1*^{134}*,ort*^{1}	Labeling clock neurons in *norpA*^{P41} and *HO* triple mutant flies with norpA rescued in different photoreceptors (with H-B eyelets laser-ablated)
	DvPdf-Gal4,UAS-GFP; HisCl1^{134}*,ort*^{1}<br>*DvPdf-Gal4,UAS-GFP; ninaE*^{I17}*,HisCl1*^{134}*,ort*^{1}<br>*Rh5*^{2}*,DvPdf-Gal4,UAS-GFP; HisCl1*^{134}*,ort*^{1}<br>*DvPdf-Gal4,UAS-GFP; Rh6*^{1}*,HisCl1*^{134}*,ort*^{1}<br>*Rh5*^{2}*,DvPdf-Gal4,UAS-GFP; Rh6*^{1}*,HisCl1*^{134}*,ort*^{1}	Labeling clock neurons in *HO* flies with loss of Rh1, Rh5, or Rh6 (with H-B eyelets laser-ablated)<br>*ninaE*^{I17}: Rhodopsin 1 mutant<br>*Rh5*^{2}: Rhodopsin 5 mutant<br>*Rh6*^{1}: Rhodopsin 6 mutant
Fig. 1e	*norpA*^{P41}*/y; Rh5-Gal4,UAS-norpA/DvPdf-LexA,LexAop-GFP*<br>*norpA*^{P41}*/y; Rh5-Gal4,UAS-norpA/DvPdf-LexA,LexAop-GFP; HisCl1*^{134}*,ort*^{1}<br>*norpA*^{P41}*/y; Rh6-Gal4,UAS-norpA/DvPdf-LexA,LexAop-GFP*<br>*norpA*^{P41}*/y; Rh6-Gal4,UAS-norpA/DvPdf-LexA,LexAop-GFP; HisCl1*^{134}*,ort*^{1}	Labeling clock neurons in *norpA*^{P41} or *norpA*^{P41} and *HO* triple mutant flies with norpA rescued in different photoreceptors (with H-B eyelets laser-ablated)
Fig. 2a	*Rh5-Gal4,Rh6-Gal4/UAS-FRT-STOP-FRT-GFP; ChAT-FLP/+*	Labeling ChAT-expressing R8s<br>ChAT: Choline acetyltransferase, which catalyzes biosynthesis of acetylcholine and is considered as a specific marker for cholinergic neurons<br>FLP/FRT: FLP recombinase can recognize FRT site and catalyze the removal of DNA segments between two FRT sites
Fig. 2b	*Rh5-Gal4/UAS-FRT-STOP-FRT-GFP; ChAT-FLP/+*<br>*Rh6-Gal4/UAS-FRT-STOP-FRT-GFP; ChAT-FLP/+*	Labeling ChAT-expressing R8s
Fig. 2c	*Rh5-Gal4/UAS-FLP; VAChT-FRT-STOP-FRT-HA/+*<br>*Rh6-Gal4/UAS-FLP; VAChT-FRT-STOP-FRT-HA/+*	HA labeling in ChAT-expressing R8s<br>VAChT: vesicular ACh transporter
Fig. 2d	*Rh5-Gal4; UAS-FRT-STOP-FRT-GFP,ChAT-FLP/+*	HA labeling in ChAT-expressing R8s

	Rh6-Gal4; UAS-FRT-STOP-FRT-GFP,ChAT-FLP/+	
Fig. 2e	*Rh5-Gal4,Rh6-Gal4; UAS-norpA*<br>*norpA*^{P41}*/y*<br>*norpA*^{P41}*/y; Rh5-Gal4,Rh6-Gal4; UAS-norpA*	norpA rescue in R8s of *norpA*^{p41} flies
Fig. 2f	*norpA*^{P41}*;; cry*^{02}<br>*norpA*^{P41}*/y; Rh5-Gal4,Rh6-Gal4,UAS-norpA; cry*^{02}	norpA rescue in R8s of *norpA*^{P41} and *cry*^{02} double mutant flies
	norpA^{P41}*/y; Rh5-Gal4,Rh6-Gal4,UAS-norpA/Rh6-hid,rpr; cry*^{02}	norpA rescue in R8s of *norpA*^{P41} and *cry*^{02}double mutant flies (with H-B eyelets ablated genetically)
Fig. 2g	*ort*^{1}	*ort*^{1}mutant flies
	norpA^{P41}*/y; Rh5-Gal4,Rh6-Gal4,UAS-norpA; ort*^{1}	norpA rescue in R8s of *norpA*^{P41} and *ort*^{1} double mutant flies
	norpA^{P41}*/y; Rh5-Gal4,Rh6-Gal4,UAS-norpA/UAS-Hdc-sgRNA,UAS-Cas9.P2*	Hdc knockout in R8s of flies with norpA rescued in R8s of *norpA*^{P41} flies<br>Cas9 and sgRNA: conditional gene knockout through CRISPR/Cas9
Fig. 2h	*norpA*^{p41}*/y; Rh5-Gal4,Rh6-Gal4,UAS-norpA; cry*^{02}*,ort*^{1}	norpA rescue in R8s of *norpA*^{P41}, *ort*^{1}, and *cry*^{02} triple mutant flies
Fig. 2i	*norpA*^{P41}*/y; Rh5-Gal4,Rh6-Gal4,UAS-norpA/UAS-ChAT-sgRNA,UAS-Cas9.P2; ort*^{1}*,cry*^{02}	ChAT knockout in R8s with norpA rescued in *norpA*^{P41}, *ort*^{1}, and *cry*^{02} triple mutant flies
Fig. 2j	*norpA*^{P41}*/y; Rh5-Gal4,Rh6-Gal4,UAS-norpA/UAS-Cas9.P2,UAS-ChAT-sgRNA*	ChAT knockout in R8s with norpA rescued in *norpA*^{P41} flies
Fig. 3a	*UAS-GFP,QUAS-tdTomato/+; Rh5-Gal4/trans-Tango; ort-QS/+*	Exclusion of QUAS-tdTomato expression in ort-expressing postsynaptic neurons of pR8s by ort-QS
	UAS-GFP,QUAS-tdTomato/+; Rh6-Gal4/trans-Tango; ort-QS/+	Exclusion of QUAS-tdTomato expression in ort-expressing postsynaptic neurons of yR8s by ort-QS
Fig. 3b	*UAS-GFP,QUAS-tdTomato/+; VT037867-LexA,LexAop-GFP,Rh5-Gal4/ trans-Tango; ort-QS/+*	AMA neurons overlap with postsynaptic neurons of pR8s
	UAS-GFP,QUAS-tdTomato/+; VT037867-LexA,LexAop-GFP/trans-Tango; Rh6-Gal4/ort-QS	AMA neurons overlap with postsynaptic neurons of yR8s
Fig. 3c	*Rh5-Gal4/VT037867-LexA; UAS-GFP*$^{1-10}$*,LexAop-GFP*^{11}*/+*<br>*Rh6-LexA/+; UAS-GFP*$^{1-10}$*,LexAop-GFP*^{11}*/VT037867-Gal4*<br>*GMR-LexA/+; UAS-GFP*$^{1-10}$*,LexAop-GFP*^{11}*/VT037867-Gal4*	GRASP between AMA neurons and pR8s, yR8s, or eye photoreceptors<br>GRASP: GFP Reconstitution Across Synaptic Partner
Fig. 3d	*VT037867-Gal4,UAS-GCaMP6m*<br>*Rh5*^{2}*; VT037867-Gal4,UAS-GCaMP6m,Rh6*^{1}<br>*norpA*^{P41}*/y; Rh5-Gal4,Rh6-Gal4/+; VT037867-Gal4,UAS-GCaMP6m/UAS-norpA*	Labeling AMA neurons in WT, or *Rh5*^{2};*Rh6*^{1} mutant flies, or *norpA*^{P41} flies with norpA rescued in R8s
Fig. 3e	*VT037867-Gal4,UAS-GCaMP6m*<br>*Rh5*^{2}*; VT037867-Gal4,UAS-GCaMP6m,Rh6*^{1}<br>*norpA*^{P41}*/y; Rh5-Gal4,Rh6-Gal4/+; VT037867-Gal4,UAS-GCaMP6m/UAS-norpA*<br>*Hdc*^{JK910}*; VT037867-Gal4,UAS-GCaMP6m*<br>*VT037867-Gal4,UAS-GCaMP6m,HisCl*^{134}*,ort*^{1}<br>*Rh5-Gal4,Rh6-Gal4,UAS-Cas9.P2/UAS-ChAT sgRNA; VT037867-Gal4,UAS-GCaMP6m*<br>*Rh5-Gal4,Rh6-Gal4,UAS-Cas9.P2/UAS-VAChT sgRNA; VT037867-Gal4,UAS-GCaMP6m*	Labeling AMA neurons in WT, or *Rh5*^{2};*Rh6*^{1} mutant flies, or *norpA*^{P41} flies with norpA rescued in R8s, or *Hdc*^{JK910}, or *ort*^{1} flies, or flies with ChAT knocked out in R8s
Fig. 3f	*27G06-LexA,LexAop-GFP*<br>*24C08-Gal4,UAS-GCaMP6m*<br>*ort-Gal4.C1a.DBD, ET.VP16.AD [tou9A30],UAS-GFP*	Labeling L1, Tm9, Tm20, respectively

	norpA^{P41}/y; 27G06-LexA,LexAop-GFP/Rh5-Gal4,Rh6-Gal4,UAS-norpA<br>norpA^{P41}/y; Rh5-Gal4,Rh6-Gal4,UAS-norpA; 24C08-Gal4,UAS-GCaMP6m<br>norpA^{P41}/y; ort-Gal4.C1a.DBD, ET.VP16.AD [tou9A30],UAS-GFP/Rh5-Gal4,Rh6-Gal4,UAS-norpA	Labeling L1, Tm9, or Tm20 in *norpA*^{P41} flies with norpA rescued in R8s
	norpA^{P41}/y; 27G06-LexA,LexAop-GFP/Rh5-Gal4,Rh6-Gal4,UAS-norpA; HisCl^{134},ort^{1}<br>norpA^{P41}/y; Rh5-Gal4,Rh6-Gal4,UAS-norpA; 24C08-Gal4,UAS-G6M,HisCl^{134},ort^{1}<br>norpA^{P41}/y; ort-Gal4.C1a.DBD, ET.VP16.AD [tou9A30],UAS-GFP/Rh5-Gal4,Rh6-Gal4,UAS-NorpA; HisCl^{134},ort^{1}	Labeling L1, Tm9, or Tm20 in *HO* and *norpA*^{P41} flies with norpA rescued in R8s
Fig. 3g	VT037867-LexA/SS00307-AD; UAS-post-GFP$^{1-10}$,LexAop-post-GFP^{11}/SS00307-DBD<br>VT037867-LexA/SS00355-AD; UAS-post-GFP$^{1-10}$,LexAop-post-GFP^{11}/SS00355-DBD	p-GRASP between AMA neurons and Tm9 or Tm20
Fig. 3h	UAS-ort/+; VT037867-Gal4,UAS-GCaMP6m,cry^{02},HisCl1^{134},ort^{1}	Ectopic ort expression in AMA neurons of *CHO* flies
Fig. 4a	VT037867-LexA,DvPdf-Gal4/LexAop2-Syb::GFP-P10,LexAop-QF2::SNAP25::HIVNES::Syntaxin; UAS-B3Recombinase,UAS<B3STOP<BoNT/A,UAS<B3STOP<BoNT/A,QUAS-mtdTomato::HA/+	Labeling presynaptic AMA neurons of clock neurons with BAcTrace
Fig. 4b	UAS-FRT-STOP-FRT-GFP/+; VT037867-Gal4/ChAT-FLP	Labeling ChAT-expressing AMA neurons
	UAS-CsChrimson/+; DvPdf-LexA,LexAop-GCaMP6m/VT037867-Gal4	Expression of CsChrimson and GCaMP in AMA and clock neurons, respectively<br>CsChrimson: red-light-gated cation channel of optogenetic activation of neurons
Fig. 4c	norpA^{P41}/y; Rh6-LexA,LexAop-norpA/UAS-TNTE;DvPdf-LexA,LexAop-GCaMP6m/VT037867-Gal4	Labeling clock neurons in *norpA*^{P41} flies with norpA rescued in R8s and with AMA neurons silenced by TNT
Fig. 4d	pBPhsFlp2::PEST/+;; UAS-FRT>STOP>FRT-myr::smGFP-HA,UAS-FRT>STOP>FRT-myr::smGFP-V5-THS-10XUAS-FRT>STOP>FRT-myr::smGFP-FLAG/VT037867-Gal4	Single-cell labeling of AMA neurons by MCFO<br>MCFO: Multi-Color-Flip-Out method for sparse-labelling
Fig. 4e	pBPhsFlp2::PEST/+;Rh5-eGFP/Rh6-LexA,LexAop-tdTomato; UAS-FRT>STOP>FRT-myr::smGFP-HA,UAS-FRT>STOP>FRT-myr::smGFP-V5-THS-10XUAS-FRT>STOP>FRT-myr::smGFP-FLAG/VT037867-Gal4	Simultaneous labeling of pR8s, yR8s, and AMA neurons
Fig. 4f	VT037867-Gal4,UAS-GCaMP6m<br>shakB^{2}/y;; VT037867-Gal4,UAS-GCaMP6m	Labeling AMA neuron in *shakB*^{2} flies<br>*ShakB*^{2}: mutant of innexin8, which constitutes the gap junction channel
Fig. 5a	cry^{02},HisCl1^{134},ort^{1}<br>Rh6-Gal4/UAS-HisCl1; cry^{02},HisCl1^{134},ort^{1}	HisCl1 expression in R8s of *CHO* flies
Fig. 5b	VT037867-Gal4,UAS-GCaMP6m<br>VT037867-LexA,LexAop2-GFP; cry^{02},ort^{1}<br>VT037867-LexA,LexAop2-GFP; cry^{02},HisCl1^{134},ort^{1}	Labeling AMA neurons in *CO* or *CHO* flies
Fig. 5c	Rh6-Gal4,UAS-HisCl1/VT037867-LexA,LexAop2-GFP; cry^{02},HisCl1^{134},ort^{1}	HisCl1 expression in yR8s of *CHO* flies
Fig. 5d	UAS-ort; VT037867-Gal4,UAS-GCaMP6m<br>UAS-ort; VT037867-Gal4,UAS-G6M,HisCl1^{134},ort^{1}	Ectopic ort expressing in AMA neurons of WT or *HO* flies
Fig. 5e	VT037867-Gal4,UAS-GCaMP6m,cry^{02},HisCl1^{134},ort^{1}	Labeling AMA neurons in *CHO* flies
Fig. 5f	VT037867-Gal4,UAS-GCaMP6m<br>VT037867-Gal4,UAS-GCaMP6m,HisCl1^{134},ort^{1}	Labeling AMA neurons in WT or *CHO* flies
Fig. 5g	cry^{02},HisCl1^{134},ort^{1}	*CHO* flies
Extended Data Fig. 1a	DvPdf-Gal4,UAS-GFP; HisCl1^{134},ort^{1}<br>norpA^{P41}; DvPdf-Gal4,UAS-GFP	Labeling clock neurons in *norpA*^{P41} single mutant flies and *CHO* triple mutant flies

	DvPdf-Gal4,UAS-GFP; cry^{02},HisCl1^{134},ort^{1}	
Extended Data Fig. 1b	Rh6-Gal4,UAS-GFP<br>Rh6-hid,rpr/+; Rh6-Gal4,UAS-GFP	Labeling yR8s in WT or flies with H-B eyelets genetically ablated
Extended Data Fig. 1c	Rh6-Gal4,UAS-GFP<br>Rh6-hid,rpr/+; Rh6-Gal4,UAS-GFP	Labeling yR8s in WT or flies with H-B eyelets genetically ablated
Extended Data Fig. 1d	DvPdf-Gal4,UAS-GFP/Rh6-eGFP; HisCl1^{134},ort^{1}	Labeling clock neurons, H-B eyelets and yR8s in *HO* flies
Extended Data Fig. 1e-g	Rh6-LexA,LexAop2-GCaMP6f	Labeling yR8s and H-B eyelets before and after laser cutting
Extended Data Fig. 1h	DvPdf-Gal4,UAS-GFP; HisCl1^{134},ort^{1}<br>DvPdf-Gal4,UAS-GFP/Rh1-Gal4,UAS-TNTE; HisCl1^{134},ort^{1}<br>DvPdf-Gal4,UAS-GFP/Rh3+4-Gal4,UAS-TNTE; HisCl1^{134},ort^{1}<br>DvPdf-Gal4,UAS-GFP/Rh5-Gal4,UAS-TNTE; HisCl1^{134},ort^{1}<br>DvPdf-Gal4,UAS-GFP/Rh6-Gal4,UAS-TNTE; HisCl1^{134},ort^{1}<br>DvPdf-Gal4,UAS-GFP/Rh5-Gal4,Rh6-Gal4,UAS-TNTE; HisCl1^{134},ort^{1}	Labeling clock neurons in *HO* flies with different photoreceptors silenced by TNT
Extended Data Fig. 2a	GMR-Gal4,UAS-GFP<br>HDC-Gal4,UAS-GFP/GMR-LexA,LexAop2-tdTomato	Co-labeling histaminergic cells and eye photoreceptors
Extended Data Fig. 2b	Rh5-Gal4/+; Rh6-Gal4,UAS-GFP/+<br>HDC-Gal4,UAS-GFP/Rh5-LexA,Rh6-LexA,LexAop2-tdTomato	Co-labeling histaminergic cells and R8s
Extended Data Fig. 2c	Rh1-Gal4/UAS-FRT-STOP-FRT-GFP; ChAT-FLP/+<br>Rh3-Gal4/UAS-FRT-STOP-FRT-GFP; ChAT-FLP/+<br>Rh4-Gal4/UAS-FRT-STOP-FRT-GFP; ChAT-FLP/+	Labeling cholinergic cells that express Rh1, Rh3, or Rh4
	vGlut-FLP/ Rh1-Gal4; UAS-FRT-STOP-FRT-GFP/+<br>vGlut-FLP/ Rh3-Gal4; UAS-FRT-STOP-FRT-GFP/+<br>vGlut-FLP/ Rh4-Gal4; UAS-FRT-STOP-FRT-GFP/+	Labeling glutamatergic cells that express Rh1, Rh3, or Rh4
	Rh1-Gal4/UAS-FRT-STOP-FRT-GFP; TH-FLP/+<br>Rh3-Gal4/UAS-FRT-STOP-FRT-GFP; TH-FLP/+<br>Rh4-Gal4/UAS-FRT-STOP-FRT-GFP; TH-FLP/+	Labeling dopaminergic cells that express Rh1, Rh3, or Rh4
	Rh1-Gal4/vGAT-LexA; UAS-FRT-STOP-FRT-CsChrimson,LexAop-FLP<br>Rh3-Gal4/vGAT-LexA; UAS-FRT-STOP-FRT-CsChrimson,LexAop-FLP<br>Rh4-Gal4/vGAT-LexA; UAS-FRT-STOP-FRT-CsChrimson,LexAop-FLP	Labeling GABAergic cells that express Rh1, Rh3, or Rh4
Extended Data Fig. 2d	vGlut-FLP/ Rh5-Gal4,Rh6-Gal4; UAS-FRT-STOP-FRT-GFP/+<br>Rh5-Gal4,Rh6-Gal4/UAS-FRT-STOP-FRT-GFP; TH-FLP/+<br>Rh5-Gal4,Rh6-Gal4/vGAT-LexA; UAS-FRT-STOP-FRT-CsChrimson,LexAop-FLP/+<br>Rh5-Gal4,Rh6-Gal4/UAS-FRT-STOP-FRT-GFP; Trh-FLP/+	Labeling R8s that contain glutamate, dopamine, GABA, or serotonin
Extended Data Fig. 3a	Rh5-Gal4,Rh6-Gal4; UAS-norpA<br>norpA^{P41}/y<br>norpA^{P41}/y; Rh5-Gal4,Rh6-Gal4/+; UAS-norpA/+	norpA rescue in R8s of *norpA*^{P41} flies
Extended Data Fig. 3b	w^{1118}<br>norpA^{P41}; cry^{02}	
	norpA^{P41}/y; Rh5-Gal4,Rh6-Gal4,UAS-norpA; cry^{02}	norpA rescue in R8s of *norpA*^{P41};;*cry*^{02} double mutant flies

	norpA^{P41}*/y; Rh5-Gal4,Rh6-Gal4,UAS-norpA/Rh6-hid,rpr; cry*^{02}	norpA rescue in R8s of *norpA*^{P41}*;;cry*^{02}double mutant flies (with H-B eyelets ablated genetically)
Extended Data Fig. 3c	*norpA*^{P41}*/y; Rh5-Gal4,Rh6-Gal4,UAS-norpA/UAS-Hdc-sgRNA,UAS-Cas9.P2*	Knocking out *hdc* in R8s with norpA rescued in *norpA*^{P41} flies
Extended Data Fig. 3d	*norpA*^{P41}*; Rh5-Gal4, Rh6-Gal4,UAS-norpA; ort*^{1}*,cry*^{02}	norpA rescue in R8s of *norpA*^{P41}*;;cry*^{02}*,ort*^{1} triple mutant flies
Extended Data Fig. 3e	*norpA*^{P41}*/y; Rh5-Gal4,Rh6-Gal4,UAS-norpA/UAS-ChAT-sgRNA,UAS-Cas9.P2; ort*^{1}*,cry*^{02}	ChAT knockout in norpA-rescued R8s of *norpA*^{P41}*;ort*^{1}*, cry*^{02} flies
Extended Data Fig. 3f	*norpA*^{P41}*/y; Rh5-Gal4,Rh6-Gal4,UAS-norpA/UAS-Cas9.P2,UAS-ChAT-sgRNA*	ChAT knockout in norpA-rescued R8s of *norpA*^{P41} flies
Extended Data Fig. 4a	*UAS-GFP,QUAS-tdTomato/+; Rh5-Gal4/trans-Tango*<br>*UAS-GFP,QUAS-tdTomato/+; Rh6-Gal4/trans-Tango*	Labeling postsynaptic neurons of pR8s and yR8s
Extended Data Fig. 4b	*UAS-GFP,QUAS-tdTomato/+; Rh5-Gal4,VT037867-LexA, LexAop-GFP/trans-Tango; ort-QS/+*<br>*UAS-GFP,QUAS-tdTomato/+; VT037867-LexA,LexAop-GFP/trans-Tango;Rh6-Gal4/ort-QS*	Co-labeling of AMA neurons and non-*ort* target of R8s
Extended Data Fig. 4c	*pBPhsFlp2::PEST/+; ; UAS-FRT>STOP>FRT-myr::smGFP-HA,UAS-FRT>STOP>FRT-myr::smGFP-V5-THS-10XUAS-FRT>STOP>FRT-myr::smGFP-FLAG/VT037867-Gal4*	Single-cell labeling of AMA neurons with MCFO
Extended Data Fig. 4d	*VT037867-Gal4, UAS-GCaMP6m*	Single-cell labeling of AMA neurons by neurobiotin injection
Extended Data Fig. 4e	*pBPhsFlp2::PEST/+; GMR-RFP/+; UAS-FRT>STOP>FRT-myr::smGFP-HA,UAS-FRT>STOP>FRT-myr::smGFP-V5-THS-10XUAS-FRT>STOP>FRT-myr::smGFP-FLAG/VT037867-Gal4*	Co-labeling of photoreceptors and single AMA neuron
Extended Data Fig. 4f	*pBPhsFlp2::PEST/+; Rh5-eGFP /+; UAS-FRT>STOP>FRT-myr::smGFP-HA,UAS-FRT>STOP>FRT-myr::smGFP-V5-THS-10XUAS-FRT>STOP>FRT-myr::smGFP-FLAG/VT037867-Gal4*	Co-labeling of pR8s and single AMA neuron
	pBPhsFlp2::PEST/+; Rh6-eGFP /+; UAS-FRT>STOP>FRT-myr::smGFP-HA,UAS-FRT>STOP>FRT-myr::smGFP-V5-THS-10XUAS-FRT>STOP>FRT-myr::smGFP-FLAG/VT037867-Gal4	Co-labeling of yR8s and single AMA neuron
Extended Data Fig. 4g	*VT037867-LexA/SS00307-AD; UAS-GFP*$^{1-10}$*,LexAop-GFP*^{11}*/SS00307-DBD*	GRASP between AMA neuron and Tm9
	VT037867-LexA/SS00355-AD; UAS-GFP$^{1-10}$*,LexAop-GFP*^{11}*/SS00355-DBD*	GRASP between AMA neuron and Tm20
Extended Data Fig. 5a,b	*UAS-sPA-GFP/+; UAS-tdTomato/VT037867-Gal4*	Labeling AMA neurons with photoactivable GFP
Extended Data Fig. 5c	*UAS-RFP,LexAop-GFP/+; SS01050-AD/VT037867-LexA; SS01050-DBD/+*	Co-labeling AMA neurons (*VT037867-LexA*) and aMe12 neurons.
Extended Data Fig. 5d	*UAS-GFP,QUAS-tdTomato/+; Rh5-Gal4/trans-Tango; ort-LexA,LexAop-GFP/+*<br>*UAS-GFP,QUAS-tdTomato/+; Rh6-Gal4/trans-Tango; ort-LexA,LexAop-GFP/+*	Co-labeling ort-expressing cells and postsynaptic neurons of pR8s or yR8s
Extended Data Fig. 5e	*UAS-FRT-STOP-FRT-CsChrimson,LexAop-FLP/SS00691-AD; ort-LexA/SS00691-DBD*<br>*UAS-FRT-STOP-FRT-CsChrimson,LexAop-FLP/ort-Gal4.C1a.DBD; ort-LexA/dVP16AD[ET18k]*	Labeling ort-expressing L1, Tm5, Tm9, or Tm20, respectively

	UAS-FRT-STOP-FRT-CsChrimson,LexAop-FLP/SS00307-AD; ort-LexA/SS00307-DBD<br>*UAS-FRT-STOP-FRT-CsChrimson,LexAop-FLP/SS00355-AD; ort-LexA/SS00355-DBD*	
Extended Data Fig. 5f	*Rh6-LexA/SS00691-AD; UAS-GFP$^{1-10}$,LexAop-GFP^{11}/SS00691-DBD*<br>*Rh6-LexA/ort-Gal4.C1a.DBD; UAS-GFP$^{1-10}$,LexAop-GFP^{11}/dVP16AD[ET18k]*<br>*Rh6-LexA/SS00307-AD; UAS-GFP$^{1-10}$,LexAop-GFP^{11}/SS00307-DBD*<br>*Rh6-LexA/SS00355-AD; UAS-GFP$^{1-10}$,LexAop-GFP^{11}/ SS00355-DBD*	GRASP between yR8s and L1, Tm5, Tm9, or Tm20, respectively
Extended Data Fig. 6a	*norpA^{P41}/y;; VT037867-Gal4,UAS-GCaMP6m/+*<br>*norpA^{P41}/y; Rh5-Gal4,Rh6-Gal4/+; VT037867-Gal4,UAS-GCaMP6m/UAS-norpA*	Labeling AMA neurons in *norpA^{P41}* flies or *norpA^{P41}* flies with norpA rescued in R8s
Extended Data Fig. 6b	*norpA^{P41}/y; 27G06-LexA,LexAop-GFP/Rh5-Gal4,Rh6-Gal4,UAS-norpA*<br>*norpA^{P41}/y; Rh5-Gal4,Rh6-Gal4,UAS-norpA; 24C08-Gal4,UAS-GCaMP6m*<br>*norpA^{P41}/y; ort-Gal4.C1a.DBD, ET.VP16.AD [tou9A30],UAS-GFP/Rh5-Gal4,Rh6-Gal4,UAS-norpA*	Labeling L1, Tm9, or Tm20 in *norpA^{P41}* flies with norpA rescued in R8s
Extended Data Fig. 6c	*27G06-LexA,LexAop-GFP*<br>*24C08-Gal4,UAS-GCaMP6m*<br>*ort-Gal4.C1a.DBD, ET.VP16.AD [tou9A30],UAS-GFP*	Labeling L1, Tm9, or Tm20
Extended Data Fig. 6e	*VT037867-LexA/SS00690-AD; UAS-post-GFP$^{1-10}$,LexAop-post-GFP^{11}/SS00690-DBD*	p-GRASP between AMA neurons and L2 neurons
Extended Data Fig. 6f	*VT037867-LexA/SS00307-AD; UAS-post-GFP$^{1-10}$,LexAop-post-GFP^{11}/SS00307-DBD*<br>*VT037867-LexA/SS00355-AD; UAS-post-GFP$^{1-10}$,LexAop-post-GFP^{11}/SS00355-DBD*	p-GRASP between AMA neurons and Tm9 and Tm20 neurons
Extended Data Fig. 6g	*VT037867-Gal4,UAS-GCaMP6m*	Labeling AMA neurons.
	UAS-ort/+; VT037867-Gal4,UAS-GCaMP6m	Labeling AMA neurons that ectopically express *ort*
Extended Data Fig. 7a,b	*trans-Tango/+; VT03867-Gal4,QUAS-tdTomato/+*	Labeling postsynaptic neurons of AMA neurons
Extended Data Fig. 7c	*trans-Tango/+; VT03867-Gal4,QUAS-tdTomato/R54D11-LexA, LexAop-GCaMP7s*	Co-labeling ITP-LNd/5th s-LNv and postsynaptic neurons of AMA neurons
Extended Data Fig. 7d	*UAS-CsChrimson/+; DvPdf-LexA,LexAop-GCaMP6m/VT037867-Gal4*	Expression of CsChrimson and GCaMP in AMA and clock neurons, respectively
Extended Data Fig. 7e	*norpA^{P41}/y; Rh5-Gal4,Rh6-Gal4,UAS-NorpA/Rh6-eGFP; DvPdf-LexA,LexAop-GCaMP6m/+*	Labeling clock neurons in *norpA^{P41}* flies with norpA rescued in R8s of (with H-B eyelets laser-ablated)
Extended Data Fig. 8a	*VT037867-Gal4,UAS-GFP*<br>*shakB^{2}/y;; VT037867-Gal4,UAS-GFP/+*<br>*UAS-GFP,QUAS-tdTomato/+; trans-Tango/+; VT037867-Gal4/+*	Labeling AMA neuron in *shakB^{2}* flies
Extended Data Fig. 8c	*UAS-GCaMP6m; VT037867-Gal4,UAS-GCaMP6m/+*	Labeling AMA neurons
Extended Data Fig. 9a	*VT037867-Gal4,UAS-GCaMP6m*<br>*VT037867-LexA,LexAop2-GFP; cry^{02},ort^{1}*<br>*VT037867-LexA,LexAop2-GFP; cry^{02},HisCl1^{134},ort^{1}*	Labeling AMA neurons in *CO* or *CHO* flies
Extended Data Fig. 9b	*VT037867-Gal4,UAS-GCaMP6m*	Labeling AMA neurons
Extended Data Fig. 9c	*norpA^{P41}/y; Rh5-Gal4,Rh6-Gal4/+; VT037867-Gal4,UAS-GCaMP6m/UAS-norpA*<br>*norpA^{P41}/y; Rh5-Gal4,Rh6-Gal4,UAS-norpA/VT037867-LexA,LexAop-GFP ; cry^{02},ort^{1}*<br>*norpA^{P41}/y; Rh5-Gal4,Rh6-Gal4,UAS-norpA/VT037867-LexA,LexAop-GFP ; cry^{02},HisCl1^{134},ort^{1}*	Labeling AMA neurons in *norpA^{P41};CHO* (or *CO*) flies with norpA rescued in R8s

Extended Data Fig. 9d	*VT037867-Gal4,UAS-GCaMP6m*<br>*VT037867-Gal4,UAS-GCaMP6m,HisCl1^{134},ort^{1}*	Labeling AMA neurons in WT or *HO* flies
Extended Data Fig. 9e	*Rh6-LexA,LexAop2-GCaMP6f*<br>*Rh6-LexA,LexAop2-GCaMP6f; cry^{02},HisCl1^{134},ort^{1}*	Labeling yR8s in WT or *CHO* flies
Extended Data Fig. 9f	*UAS-ort; VT037867-Gal4,UAS-GCaMP6m*	Ectopic *ort* expression in AMA neurons
Extended Data Fig. 10a,b	*DvPdf-Gal4,UAS-GFP; cry^{02},HisCl1^{134},ort^{1}*	Labeling clock neurons in *CHO* flies
Extended Data Fig. 10c	*w^{1118}*	Re-entrainment in wild type flies.

Supplementary Table 2 | Statistical details

Figure		Cell type	P value
Fig. 1b	one-way ANOVA followed by Tukey's post hoc test	l-LNv	WT (n = 9 from 5 flies) vs. HO (n = 11 from 7 flies), P = 2.0690E-6<br>WT (n = 9 from 5 flies) vs. HDC (n = 11 from 7 flies), P = 0.0078<br>HO (n = 11 from 7 flies) vs. HDC (n = 11 from 7 flies), P = 0.0317
		s-LNv	WT (n = 8 from 5 flies) vs. HO (n = 8 from 5 flies), P = 0.0049<br>WT (n = 8 from 5 flies) vs. HDC (n = 6 from 6 flies), P = 0.0215<br>HO (n = 8 from 7 flies) vs. HDC (n = 8 from 8 flies), P = 0.8511
		ITP-LNd	WT (n = 8 from 6 flies) vs. HO (n = 7 from 6 flies), P = 0.0001<br>WT (n = 8 from 6 flies) vs. HDC (n = 6 from 5 flies), P = 0.0026<br>HO (n = 7 from 6 flies) vs. HDC (n = 6 from 5 flies), P = 0.5360
		5th s-LNv	WT (n = 6 from 6 flies) vs. HO (n = 8 from 7 flies), P = 4.3027E-5<br>WT (n = 6 from 6 flies) vs. HDC (n = 8 from 8 flies), P = 1.0067E-5<br>HO (n = 8 from 7 flies) vs. HDC (n = 8 from 8 flies), P = 0.7404
Fig. 1c	one-way ANOVA followed by Tukey's post hoc test	l-LNv	HO (n = 11 from 7 flies) vs. HO+norpA^{P41} (n = 5 from 5 flies), P = 2.2389E-8<br>HO (n = 11 from 7 flies) vs. HO+eye removal (n = 5 from 5 flies), P = 2.2389E-8<br>HO (n = 11 from 7 flies) vs. HO+Rh6-hid,rpr (n = 16 from 10 flies), P = 0.0006<br>HO (n = 11 from 7 flies) vs. HO+H-B ablation (n = 6 from 6 flies), P = 0.0005<br>HO+Rh6-hid,rpr (n = 16 from 10 flies) vs. HO+H-B ablation (n = 6 from 6 flies), P = 0.5321
		s-LNv	HO (n = 8 from 5 flies) vs. HO+norpA^{P41} (n = 6 from 5 flies), P = 0.0139<br>HO (n = 8 from 5 flies) vs. HO+eye removal (n = 6 from 5 flies), P = 0.0139<br>HO (n = 8 from 5 flies) vs. HO+Rh6-hid,rpr (n = 10 from 6 flies), P = 0.0207<br>HO (n = 8 from 5 flies) vs. HO+H-B ablation (n = 9 from 6 flies), P = 0.0385<br>HO+Rh6-hid,rpr (n = 10 from 6 flies) vs. HO+H-B ablation (n = 9 from 6 flies), P = 0.9855
		ITP-LNd	HO (n = 7 from 6 flies) vs. HO+norpA^{P41} (n = 5 from 5 flies), P = 0<br>HO (n = 7 from 6 flies) vs. HO+eye removal (n = 5 from 5 flies), P = 0<br>HO (n = 7 from 6 flies) vs. HO+Rh6-hid,rpr (n = 11 from 9 flies), P = 0.9968<br>HO (n = 7 from 6 flies) vs. HO+H-B ablation (n = 6 from 6 flies), P = 0.1764<br>HO+Rh6-hid,rpr (n = 11 from 9 flies) vs. HO+H-B ablation (n = 6 from 6 flies), P = 0.1467
		5th s-LNv	HO (n = 8 from 7 flies) vs. HO+norpA^{P41} (n = 5 from 5 flies), P = 6.9374E-6<br>HO (n = 8 from 7 flies) vs. HO+eye removal (n = 5 from 5 flies), P = 6.9346E-6<br>HO (n = 8 from 7 flies) vs. HO+Rh6-hid,rpr (n = 10 from 10 flies), P = 0.3838<br>HO (n = 8 from 7 flies) vs. HO+H-B ablation (n = 6 from 6 flies), P = 0.8349<br>HO+Rh6-hid,rpr (n = 10 from 10 flies) vs. HO+H-B ablation (n = 6 from 6 flies), P = 0.7999
Fig. 1d-middle	Kruskal-Wallis followed by Dunn's tests	l-LNv	HO+norpA^{P41} (n = 5 from 5 flies) vs. HO+norpA^{P41}+Rh1>norpA rescue (n = 7 from 5 flies), P = 1<br>HO+norpA^{P41} (n = 5 from 5 flies) vs. HO+norpA^{P41}+Rh3>norpA rescue (n = 9 from 5 flies), P = 1<br>HO+norpA^{P41} (n = 5 from 5 flies) vs. HO+norpA^{P41}+Rh4>norpA rescue (n = 8 from 6 flies), P = 1<br>HO+norpA^{P41} (n = 5 from 5 flies) vs. HO+norpA^{P41}+Rh5>norpA rescue (n = 6 from 6 flies), P = 0.0111<br>HO+norpA^{P41} (n = 5 from 5 flies) vs. HO+norpA^{P41}+Rh6>norpA rescue (n = 6 from 6 flies), P = 0.0165
		s-LNv	HO+norpA^{P41} (n = 6 from 5 flies) vs. HO+norpA^{P41}+Rh1>norpA rescue (n = 6 from 5 flies), P = 1

			HO+norpA^{P41} (n = 6 from 5 flies) vs. HO+norpA^{P41}+Rh3>norpA rescue (n = 8 from 6 flies), P = 1<br>HO+norpA^{P41} (n = 6 from 5 flies) vs. HO+norpA^{P41}+Rh4>norpA rescue (n = 7 from 6 flies), P = 1<br>HO+norpA^{P41} (n = 6 from 5 flies) vs. HO+norpA^{P41}+Rh5>norpA rescue (n = 5 from 5 flies), P = 0.0251<br>HO+norpA^{P41} (n = 6 from 5 flies) vs. HO+norpA^{P41}+Rh6>norpA rescue (n = 6 from 6 flies), P = 0.0059
		ITP-LNd	HO+norpA^{P41} (n = 5 from 5 flies) vs. HO+norpA^{P41}+Rh1>norpA rescue (n = 6 from 6 flies), P = 1<br>HO+norpA^{P41} (n = 5 from 5 flies) vs. HO+norpA^{P41}+Rh3>norpA rescue (n = 9 from 7 flies), P = 1<br>HO+norpA^{P41} (n = 5 from 5 flies) vs. HO+norpA^{P41}+Rh4>norpA rescue (n = 8 from 6 flies), P = 1<br>HO+norpA^{P41} (n = 5 from 5 flies) vs. HO+norpA^{P41}+Rh5>norpA rescue (n = 7 from 6 flies), P = 0.0028<br>HO+norpA^{P41} (n = 5 from 5 flies) vs. HO+norpA^{P41}+Rh6>norpA rescue, P = 0.0034
		5th s-LNv	HO+norpA^{P41} (n = 5 from 5 flies) vs. HO+norpA^{P41} +Rh1>norpA rescue (n = 6 from 6 flies), P = 1<br>HO+norpA^{P41} (n = 5 from 5 flies) vs. HO+norpA^{P41}+Rh3>norpA rescue (n = 8 from 7 flies), P = 1<br>HO+norpA^{P41} (n = 5 from 5 flies) vs. HO+norpA^{P41}+Rh4>norpA rescue (n = 6 from 6 flies), P = 1<br>HO+norpA^{P41} (n = 5 from 5 flies) vs. HO+norpA^{P41}+Rh5>norpA rescue (n = 6 from 6 flies), P = 0.0080<br>HO+norpA^{P41} (n = 5 from 5 flies) vs. HO+norpA^{P41}+Rh6>norpA rescue (n = 6 from 6 flies), P = 0.0028
Fig. 1d-right	Kruskal-Wallis followed by Dunn's tests	l-LNv	HO (n = 11 from 7 flies) vs. HO+ninaE^{I17} (n = 7 from 5 flies), P = 1<br>HO (n = 11 from 7 flies) vs. HO+Rh5^{2} (n = 7 from 5 flies), P = 0.8486<br>HO (n = 11 from 7 flies) vs. HO+Rh6^{1} (n = 7 from 6 flies), P = 0.0117<br>HO (n = 11 from 7 flies) vs. HO+ Rh5^{2} + Rh6^{1} (n = 8 from 7 flies), P = 1.9042E-5
		s-LNv	HO (n = 8 from 5 flies) vs. HO+ninaE^{I17} (n = 8 from 5 flies), P = 1<br>HO (n = 8 from 5 flies) vs. HO+Rh5^{2} (n = 7 from 5 flies), P = 1<br>HO (n = 8 from 5 flies) vs. HO+Rh6^{1} (n = 6 from 5 flies), P = 1<br>HO (n = 8 from 5 flies) vs. HO+ Rh5^{2} + Rh6^{1} (n = 7 from 7 flies), P = 0.0075
		ITP-LNd	HO (n = 7 from 6 flies) vs. HO+ninaE^{I17} (n = 6 from 6 flies), P = 0.9665<br>HO (n = 7 from 6 flies) vs. HO+Rh5^{2} (n = 8 from 7 flies), P = 0.0064<br>HO (n = 7 from 6 flies) vs. HO+Rh6^{1} (n = 8 from 6 flies), P = 0.0130<br>HO (n = 7 from 6 flies) vs. HO+ Rh5^{2} + Rh6^{1} (n = 7 from 7 flies), P = 0.0002
		5th s-LNv	HO (n = 8 from 7 flies) vs. HO+ninaE^{I17} (n = 7 from 6 flies), P = 0.8332<br>HO (n = 8 from 7 flies) vs. HO+Rh5^{2} (n = 8 from 8 flies), P = 0.2061<br>HO (n = 8 from 7 flies) vs. HO+Rh6^{1} (n = 8 from 8 flies), P = 0.0364<br>HO (n = 8 from 7 flies) vs. HO+ Rh5^{2} + Rh6^{1} (n = 7 from 7 flies), P = 3.9085E-8
Fig. 1e	Two-tailed Mann-Whitney test	l-LNv	Rh5>norpA rescue (n = 9 from 6 flies) vs. Rh5>norpA rescue+HO (n = 6 from 6 flies), P = 0.7529
			Rh6>norpA rescue (n = 9 from 6 flies) vs. Rh6>norpA rescue+HO (n = 6 from 6 flies), P = 0.0875
		s-LNv	Rh5>norpA rescue (n = 6 from 6 flies) vs. Rh5>norpA rescue+HO (n = 5 from 5 flies), P = 0.8923
			Rh6>norpA rescue (n = 6 from 6 flies) vs. Rh6>norpA rescue+HO (n = 6 from 6 flies), P = 0.2979
		ITP-LNd	Rh5>norpA rescue (n = 6 from 6 flies) vs. Rh5>norpA rescue+HO (n = 7 from 6 flies), P = 0.7210

			Rh6>norpA rescue (n = 6 from 6 flies) vs. Rh6>norpA rescue+HO (n = 6 from 6 flies), P = 0.9383
		5th s-LNv	Rh5>norpA rescue (n = 11 from 8 flies) vs. Rh5>norpA rescue+HO (n = 8 from 6 flies), P = 0.8365
			Rh6>norpA rescue (n = 7 from 6 flies) vs. Rh6>norpA rescue+HO (n = 6 from 6 flies), P = 0.7594
Fig. 2a		ChAT+ R8s	Similar results were observed in 6 out of 6 flies.
Fig. 2b		ChAT+ yR8s	Similar results were observed in 5 out of 5 flies.
		ChAT+ pR8s	Similar results were observed in 5 out of 5 flies.
Fig. 2c		VAChT+ yR8s	Similar results were observed in 7 out of 7 flies.
		VAChT+ pR8s	Similar results were observed in 6 out of 6 flies.
Fig. 2d		ChAT+ LOVIT+ yR8s	Similar results were observed in 6 out of 6 flies.
		ChAT+ LOVIT+ pR8s	Similar results were observed in 9 out of 9 flies.
Fig. 3e	one-way ANOVA followed by Tukey's post hoc test	AMA neurons	WT (n = 15 from 10 flies) vs. Rh5^{2}; Rh6^{1}(n = 10 from 6 flies), P = 3.1849E-8<br>WT (n = 15 from 10 flies) vs. R8>norpA rescue(n = 7 from 6 flies), P = 0.9997<br>WT (n = 15 from 10 flies) vs. HisCl1^{134},ort^{1} (n = 6 from 5 flies), P = 0.0775<br>WT (n = 15 from 10 flies) vs. CIM (n = 7 from 7 flies), P = 0.9679<br>WT (n = 15 from 10 flies) vs. Hdc^{JK910} (n = 6 from 5 flies), P = 0.1768<br>WT (n = 15 from 10 flies) vs. HisCl1^{134},ort^{1} (n = 6 from 5 flies), P = 0.1015<br>WT (n = 15 from 10 flies) vs. MCA (n = 11 from 10 flies), P = 2.8524E-8<br>WT (n = 15 from 10 flies) vs. ChAT CKO (n = 11 from 6 flies), P = 3.8396E-8<br>WT (n = 15 from 10 flies) vs. VAChT CKO (n = 7 from 6 flies), P = 2.8524E-8
Fig. 3f	one-way ANOVA followed by Tukey's post hoc test	Tm20	WT (n = 10 from 9 flies) vs. R8>norpA rescue (n = 5 from 5 flies), P = 0.0163<br>WT (n = 10 from 9 flies) vs. R8>norpA rescue+HO (n = 10 from 8 flies), P = 0<br>WT (n = 10 from 9 flies) vs. R8>norpA rescue+HO+MCA (n = 5 from 5 flies), P = 7.7304E-7<br>R8>norpA rescue (n = 5 from 5 flies) vs. R8>norpA rescue+HO (n = 10 from 8 flies), P = 2.8556E-7<br>R8>norpA rescue (n = 5 from 5 flies) vs. R8>norpA rescue+HO+MCA (n = 5 from 5 flies), P = 2.2327E-5<br>R8>norpA+HO rescue (n = 10 from 8 flies) vs. R8>norpA rescue+HO+MCA (n = 5 from 5 flies), P = 0.0058
		L1	WT (n = 9 from 8 flies) vs. R8>norpA rescue (n = 5 from 5 flies), P = 0.0002<br>WT (n = 9 from 8 flies) vs. R8>norpA rescue+HO (n = 10 from 9 flies), P = 1.9808E-7<br>WT (n = 9 from 8 flies) vs. R8>norpA rescue+HO+MCA (n = 5 from 5 flies), P = 4.3755E-8<br>R8>norpA rescue (n = 5 from 5 flies) vs. R8>norpA rescue+HO (n = 10 from 9 flies), P = 0.0003<br>R8>norpA rescue (n = 5 from 5 flies) vs. R8>norpA rescue+HO+MCA (n = 5 from 5 flies), P = 0.0126<br>R8>norpA+HO rescue (n = 10 from 9 flies) vs. R8>norpA rescue+HO+MCA (n = 5 from 5 flies), P = 0.7130

		Tm9	WT (n = 6 from 6 flies) vs. R8>norpA rescue (n = 6 from 6 flies), P = 1.7871E-8<br>WT (n = 6 from 6 flies) vs. R8>norpA rescue+HO (n = 6 from 6 flies), P = 0<br>WT (n = 6 from 6 flies) vs. R8>norpA rescue+HO+MCA (n = 6 from 6 flies), P = 0<br>R8>norpA rescue (n = 6 from 6 flies) vs. R8>norpA rescue+HO (n = 6 from 6 flies), P = 8.0605E-6<br>R8>norpA rescue (n = 6 from 6 flies) vs. R8>norpA rescue+HO+MCA (n = 6 from 6 flies), P = 0.0001<br>R8>norpA+HO rescue (n = 6 from 6 flies) vs. R8>norpA rescue+HO+MCA (n = 6 from 6 flies), P = 0.4202
Fig. 3h	one-way ANOVA followed by Tukey's post hoc test	AMA neurons	Depolarization after application of CIM (n = 6 from 6 flies)<br>hyperpolarization after application of MCA (n = 6 from 6 flies)<br>no response after application of both CIM and MCA (n = 6 from 6 flies).<br>CIM vs. MCA, P = 0<br>CIM vs. CIM+MCA, P = 1.4796E-6<br>MCA vs. CIM+MCA, P = 8.5592E-5
Fig. 4b	two-tailed paired Student's t-tests	AMA-driven EPSCs in clock neurons before and after MCA application	5th s-LNv, before vs. after (n = 9 from 9 flies), P = 9.5115E-7<br>ITP-LNd, before vs. after (n = 7 from 7 flies), P = 1.2697E-5<br>l-LNv, before vs. after (n = 7 from 7 flies), P = 0.0024<br>s-LNv, before vs. after (n = 6 from 6 flies), P = 0.0012
Fig. 4c		Light response of clock neurons after silence AMA neurons in R8>norpA rescue flies	5th s-LNv (n = 7 from 7 flies)<br>ITP-LNd (n = 7 from 7 flies)<br>l-LNv (n = 7 from 5 flies)<br>s-LNv (n = 7 from 6 flies)
Fig. 4d		Branch numbers of AMA neurons	Single cell (n = 7 from 6 flies)<br>Overlay (n = 7 from 7 flies)
Fig. 4e	two-tailed paired Student's t-tests	Number of columns innervated by pR8s and yR8s	pR8 vs. yR8 (n = 6 from 6 flies), P = 0.0106
Fig. 4f	two-tailed paired Student's t-tests	Pairs of AMA neurons	WT, depolarization, before vs. after $CdCl_2$ application (n = 7 from 7 flies), P = 0.0024<br>WT, hyperpolarization, before vs. after $CdCl_2$ application (n = 7 from 7 flies), P = 0.8987<br>shakB^2, depolarization, before vs. after MCA application (n = 7 from 7 flies), P = 2.6477E-5
	two-tailed unpaired Student's t-tests	Pairs of AMA neurons	WT (n = 7 from 7 flies) vs. shakB^2 (n = 7 from 7 flies),depolarization, P = 0.0031
Fig. 5b	one-way ANOVA followed by Tukey's post hoc test	AMA neurons	Peak response under current clamp:<br>WT (n = 8 from 8 flies) vs. CO (n = 7 from 7 flies), P = 0.9376<br>WT (n = 8 from 8 flies) vs. CHO (n = 10 from 10 flies), P = 0.3570<br>CO (n = 7 from 7 flies) vs. CHO (n = 10 from 10 flies), P = 0.5906<br><br>Steady response under current clamp:<br>WT (n = 8 from 8 flies) vs. CO (n = 7 from 7 flies), P = 0.0971<br>WT (n = 8 from 8 flies) vs. CHO (n = 10 from 10 flies), P = 0<br>CO (n = 7 from 7 flies) vs. CHO (n = 10 from 10 flies), P = 0<br><br>Spike firing rate under current clamp:<br>WT (n = 8 from 8 flies)<br>CO (n = 7 from 7 flies)<br>CHO (n = 10 from 10 flies)<br><br>Peak response under voltage clamp:

			WT (n = 8 from 8 flies) vs. CO (n = 8 from 8 flies), P = 0.9487<br>WT (n = 8 from 8 flies) vs. CHO (n = 8 from 8 flies), P = 0.0008<br>CO (n = 8 from 8 flies) vs. CHO (n = 8 from 8 flies), P = 0.0017<br><br>Steady response under voltage clamp:<br>WT (n = 8 from 8 flies) vs. CO (n = 8 from 8 flies), P = 0.3380<br>WT (n = 8 from 8 flies) vs. CHO (n = 8 from 8 flies), P = 0<br>CO (n = 8 from 8 flies) vs. CHO (n = 8 from 8 flies), P = 0
Fig. 5c	two-tailed unpaired Student's t-tests	AMA neurons	Peak response under current clamp:<br>CHO (n = 5 from 5 flies) vs. R8>HisCl1 rescue+CHO (n = 5 from 5 flies), P = 0.5563<br><br>Steady response under current clamp:<br>CHO (n = 5 from 5 flies) vs. R8>HisCl1 rescue (n = 6 from 6 flies), P = 0.0187<br><br>Peak response under voltage clamp:<br>CHO (n = 8 from 8 flies) vs. R8>HisCl1 rescue (n = 5 from 5 flies), P = 0.0009<br><br>Steady response under voltage clamp:<br>CHO (n = 8 from 8 flies) vs. R8>HisCl1 rescue (n = 5 from 5 flies), P = 0.0044<br><br>Spike firing rate:<br>CHO (n = 10 from 10 flies)<br>R8>HisCl1 rescue (n = 5 from 5 flies)
Fig. 5d	two-tailed unpaired Student's t-tests	AMA neurons	Peak response:<br>AMA>ort (n = 5 from 5 flies) vs. AMA>ort+HO (n = 5 from 5 flies), P = 0.5552<br>Steady response:<br>AMA>ort (n = 5 from 5 flies) vs. AMA>ort+HO (n = 5 from 5 flies), P = 0.0044<br>Steady/Peak Ratio:<br>AMA>ort (n = 5 from 5 flies) vs. AMA>ort+HO (n = 5 from 5 flies), P = 0.0003
Fig. 5e	one-way ANOVA followed by Tukey's post hoc test	AMA neurons	ACh response before, during and after light stimuli (n = 6 from 6 flies):<br>Before vs. during, P = 0.9986<br>During vs. after, P = 0.9584<br>Before vs. after, P = 0.9720
Fig. 5f		AMA neurons	Normalized response of AMA neurons:<br>WT (n = 5 from 5 flies)<br>HO (n = 5 from 5 flies)
Extended Fig. 1a	one-way ANOVA followed by Tukey's post hoc test	lLNv	HO (n = 11 from 7 flies) vs. CHO (n = 6 from 4 flies), P = 0.7261<br>HO (n = 11 from 7 flies) vs. $norpA^{P41}$ (n = 4 from 4 flies), P = 2.1406E-5<br>CHO (n = 6 from 4 flies) vs. $norpA^{P41}$ (n = 4 from 4 flies), P = 0.0004
		sLNv	HO (n = 8 from 5 flies) vs. CHO (n = 6 from 5 flies), P = 0.9824<br>HO (n = 8 from 5 flies) vs. $norpA^{P41}$ (n = 6 from 5 flies), P = 0.0369<br>CHO (n = 6 from 5 flies) vs. $norpA^{P41}$ (n = 6 from 5 flies), P = 0.0375
		5th s-LNv	HO (n = 8 from 7 flies) vs. CHO (n = 7 from 7 flies), P = 0.8312<br>HO (n = 8 from 7 flies) vs. $norpA^{P41}$ (n = 5 from 5 flies), P = 4.6691E-6<br>CHO (n = 7 from 7 flies) vs. $norpA^{P41}$ (n = 5 from 5 flies), P = 2.0277E-5
		ITP-LNd	HO (n = 7 from 6 flies) vs. CHO (n = 7 from 7 flies), P = 0.7084

			HO (n = 7 from 6 flies) vs. $norpA^{P41}$ (n = 5 from 5 flies), P = 7.7245E-6<br>CHO (n = 7 from 7 flies) vs. $norpA^{P41}$ (n = 5 from 5 flies), P = 1.8913E-6
Extended Fig. 1c		probability of H-B existence	WT:<br>Day1 (n = 12 from 7 flies)<br>Day 3 (n = 10 from 6 flies)<br>Day 7 (n = 13 from 7 flies)<br>Day 14 (n = 11 from 7 flies)<br>Rh6-hid,rpr:<br>Day 1 (n = 12 from 10 flies)<br>Day 3 (n = 11 from 8 flies)<br>Day 7 (n = 12 from 8 flies)<br>Day 14 (n = 10 from 6 flies)
		Counts of yR8s	WT:<br>Day 1 (n = 9 from 9 flies)<br>Day 3 (n = 9 from 9 flies)<br>Day 7 (n = 10 from 9 flies)<br>Day 14 (n = 7 from 7 flies)<br>Rh6-hid,rpr:<br>Day 1 (n = 9 from 8 flies)<br>Day 3 (n = 7 from 7 flies)<br>Day 7 (n = 10 from 8 flies)<br>Day 14 (n = 6 from 6 flies)
Extended Fig. 1d	two-tailed paired Student's t-tests	Pairs of clock neurons before and after H-B ablation	lLNv, before vs. after (n = 6 from 6 flies), P = 9.3569E-6<br>sLNv, before vs. after (n = 6 from 6 flies), P = 0.0126<br>5th s-LNv, before vs. after (n = 6 from 6 flies), P = 0.0931<br>ITP-LNd, before vs. after (n = 6 from 6 flies), P = 0.7236
Extended Fig. 1g	two-tailed paired Student's t-tests	Light response of H-B eyelets and yR8s	Calcium response of H-B eyelets and yR8s before and after laser-cutting of H-B eyelets.<br>yR8s (n=5 from 5flies): before vs. after laser-cutting, P = 0.1073<br>H-B eyelets (n=5 from 5flies): before vs. after laser-cutting, P = 0.0288
Extended Fig. 1h	Kruskal-Wallis followed by Dunn's tests	l-LNv	HO (n = 11 from 7 flies) vs. HO+Rh1>TNT (n = 8 from 4 flies), P = 0.9784<br>HO (n = 11 from 7 flies) vs. HO+Rh34>TNT (n = 6 from 6 flies), P = 0.9974<br>HO (n = 11 from 7 flies) vs. HO+Rh5>TNT (n = 7 from 4 flies), P = 0.5179<br>HO (n = 11 from 7 flies) vs. HO+Rh6>TNT (n = 8 from 5 flies), P = 4.3338E-6<br>HO (n = 11 from 7 flies) vs. HO+Rh56>TNT (n = 8 from 5 flies), P = 1.9868E-8
		s-LNv	HO (n = 8 from 5 flies) vs. HO+Rh1>TNT (n = 8 from 6 flies), P = 0.9997<br>HO (n = 8 from 5 flies) vs. HO+Rh34>TNT (n = 6 from 6 flies), P = 0.9947<br>HO (n = 8 from 5 flies) vs. HO+Rh5>TNT (n = 7 from 6 flies), P = 0.2097<br>HO (n = 8 from 5 flies) vs. HO+Rh6>TNT (n = 6 from 5 flies), P = 0.1762<br>HO (n = 8 from 5 flies) vs. HO+Rh56>TNT (n = 7 from 6 flies), P = 0.0049
		ITP-LNd	HO (n = 7 from 6 flies) vs. HO+Rh1>TNT (n = 7 from 6 flies), P = 0.9701<br>HO (n = 8 from 5 flies) vs. HO+Rh34>TNT (n = 6 from 6 flies), P = 0.9911<br>HO (n = 8 from 5 flies) vs. HO+Rh5>TNT (n = 9 from 7 flies), P = 0.0238<br>HO (n = 8 from 5 flies) vs. HO+Rh6>TNT (n = 10 from 8 flies), P = 0.0018<br>HO (n = 8 from 5 flies) vs. HO+Rh56>TNT (n = 6 from 6 flies), P = 5.8113E-7
		5th s-LNv	HO (n = 8 from 7 flies) vs. HO+Rh1>TNT (n = 7 from 6 flies), P = 0.9398

		HO (n = 8 from 7 flies) vs. HO+Rh34>TNT (n = 6 from 6 flies), P = 0.9998<br>HO (n = 8 from 7 flies) vs. HO+Rh5>TNT (n = 8 from 7 flies), P = 0.0645<br>HO (n = 8 from 7 flies) vs. HO+Rh6>TNT (n = 10 from 9 flies), P = 0.0137<br>HO (n = 8 from 7 flies) vs. HO+Rh56>TNT (n = 6 from 6 flies), P = 2.1670E-7
Extended Fig. 2a	LOVIT+ PRs	Similar results were observed in 5 out of 5 flies.
	Hdc+ PRs	Similar results were observed in 3 out of 3 flies.
Extended Fig. 2b	LOVIT+ R8s	Similar results were observed in 6 out of 6 flies.
	Hdc+ R8s	Similar results were observed in 4 out of 4 flies.
Extended Fig. 2c	Rh1 intersection	Rh1&ChAT: similar results were observed in 4 out of 4 flies.<br>Rh1&vGlut: similar results were observed in 3 out of 3 flies.<br>Rh1&TH: similar results were observed in 3 out of 3 flies.<br>Rh1&vGAT: similar results were observed in 4 out of 4 flies.
	Rh3 intersection	Rh3&ChAT: similar results were observed in 4 out of 4 flies.<br>Rh3&vGlut: similar results were observed in 4 out of 4 flies.<br>Rh3&TH: similar results were observed in 3 out of 3 flies.<br>Rh3&vGAT: similar results were observed in 4 out of 4 flies.
	Rh4 intersection	Rh4&ChAT: similar results were observed in 3 out of 3 flies.<br>Rh4&vGlut: similar results were observed in 3 out of 3 flies.<br>Rh4&TH: similar results were observed in 3 out of 3 flies.<br>Rh4&vGAT: similar results were observed in 4 out of 4 flies.
Extended Fig. 2d	R8 intersection	R8(Rh5+Rh6)&vGlut: similar results were observed in 3 out of 3 flies.<br>R8&TH: similar results were observed in 3 out of 3 flies.<br>R8&Trh: similar results were observed in 3 out of 3 flies.<br>R8&vGAT: similar results were observed in 4 out of 4 flies.
Extended Fig. 4a	postsynaptic target of pR8s	similar results were observed in 11 out of 11 flies.
	postsynaptic target of yR8s	similar results were observed in 19 out of 19 flies.
Extended Fig. 4b	Non-ort target of pR8s	similar results were observed in 6 out of 6 flies.
	Non-ort target of yR8s	similar results were observed in 7 out of 7 flies.
Extended Fig. 4c	Sparse labelling of AMA neurons	Similar single AMA neurons were labeled in 8 out of 37 flies.
Extended Fig. 4d	Neurobiotin-labelling of AMA neurons	Similar results were observed in 11 out of 11 flies.
Extended Fig. 4e	Colocalization of PRs and AMA neurons	Similar results were observed in 8 out of 8 samples that single AMA neurons were successfully labeled.
Extended Fig. 4f	Colocalization of pR8s and AMA neurons	Similar results were observed in 6 out of 6 flies.
	Colocalization of yR8s and AMA neurons	Similar results were observed in 5 out of 5 flies.
Extended Fig. 5b		Photo-activated: n = 10 from 10 flies<br>No photo-activation: n=7 from 7 flies
Extended Fig. 5c	Colocalization of AMA and aMe 12 neurons	Similar results were observed in 8 out of 8 flies.
Extended Fig. 5d	Ort+ target of pR8s	Similar results were observed in 6 out of 6 flies.
	Ort+ target of yR8s	Similar results were observed in 7 out of 7 flies.
Extended Fig. 5e	Ort expression of L/Tm neurons	L1: Similar results were observed in 3 out of 3 flies.<br>Tm5: Similar results were observed in 4 out of 4 flies.<br>Tm9: Similar results were observed in 7 out of 7 flies.<br>Tm20: Similar results were observed in 4 out of 4 flies.

Extended Fig. 5f		GRASP between yR8s and L/Tm neurons	Rh6&L1: Similar results were observed in 3 out of 3 flies.<br>Rh6&Tm5: Similar results were observed in 3 out of 3 flies.<br>Rh6&Tm9: Similar results were observed in 4 out of 4 flies.<br>Rh6&Tm20: Similar results were observed in 3 out of 3 flies.
Extended Fig. 6a	two-tailed paired Student's t-tests	Light response of AMA neurons	norpA^{P41} (n=7 from 7 flies) vs. R8>norpA rescue (n = 7 from 7 flies), P = 5.8591E-6
Extended Fig. 6b	one-way ANOVA followed by Tukey's post hoc test	L1	R8>norpA rescue (n = 5 from 5 flies) vs. CIM (n = 10 from 8 flies), P = 0<br>R8>norpA rescue (n = 6 from 6 flies) vs. CIM+MCA (n = 5 from 5 flies), P = 2.9141E-6<br>CIM (n = 10 from 8 flies) vs. CIM+MCA (n = 5 from 5 flies), P = 0.0361
		Tm9	R8>norpA rescue (n = 6 from 6 flies) vs. CIM (n = 5 from 5 flies), P = 0<br>R8>norpA rescue (n = 6 from 6 flies) vs. CIM+MCA (n = 6 from 6 flies), P = 0<br>CIM (n = 5 from 5 flies) vs. CIM+MCA (n = 6 from 6 flies), P = 0.0311
		Tm20	R8>norpA rescue (n = 5 from 5 flies) vs. CIM (n = 10 from 7 flies), P = 0<br>R8>norpA rescue (n = 5 from 5 flies) vs. CIM+MCA (n = 5 from 5 flies), P = 1.5230E-5<br>CIM (n = 10 from 7 flies) vs. CIM+MCA (n = 5 from 5 flies), P = 0.0062
Extended Fig. 6c	one-way ANOVA followed by Tukey's post hoc test	L1	n = 6 from 6 flies<br>Before vs. during CIM, P = 0<br>Before vs. after CIM, P = 0.4635<br>During vs. after CIM, P = 0
		Tm9	n = 6 from 6 flies<br>Before vs. during CIM, P = 2.5508E-7<br>Before vs. after CIM, P = 0.8700<br>During vs. after CIM, P = 5.7548E-7
		Tm20	n = 6 from 6 flies<br>Before vs. during CIM, P = 2.3678E-7<br>Before vs. after CIM, P = 0.5633<br>During vs. after CIM, P = 1.2360E-6
Extended Fig. 6e		GRASP between AMA and L2	Similar results were observed in 6 out of 6 flies.
Extended Fig. 6f		p-GRASP between AMA and Tm neurons	AMA&Tm20: Similar results were observed in 9 out of 9 flies.<br>AMA&Tm9: Similar results were observed in 4 out of 4 flies.
Extended Fig. 6g	Two-tailed Mann-Whitney tests	AMA neurons	WT (n = 6 from 4 flies) vs. VT>ort (n = 6 from 5 flies), P = 0.0028
Extended Fig. 7a&b		Colabelling of AMA>Tango, anti-TIM, and anti-PDF	Similar results were observed in 20 out of 20 flies.
Extended Fig. 7c		Colabelling of AMA>Tango and R54D11-LexA	Similar results were observed in 13 out of 13 flies.
Extended Fig. 7d	two-tailed paired Student's t-tests	Pairs of clock neurons	ITP-LNd vs. 5th s-LNv (n = 6 from 6 flies), P = 0.0017<br>ITP-LNd vs. l-LNv (n = 7 from 7 flies), P = 0.0104<br>ITP-LNd vs. s-LNv (n = 6 from 6 flies), P = 0.0031
Extended Fig. 7e			l-LNv: n = 7 from 5 flies<br>s-LNv: n = 7 from 5 flies<br>ITP-LNd: n = 7 from 6 flies<br>5th s-LNv: n = 9 from 9 flies

Extended Fig. 8a		Connection among AMA neurons	Neurobiotin-injection in WT: similar results were observed in 11 out of 11 flies.<br>Neurobiotin-injection in *shakB*^{2}: similar results were observed in 3 out of 3 flies.<br>AMA>Tango: similar results were observed in 6 out of 6 flies.
Extended Fig. 8c		Calcium response of AMA neurons	n = 6 from 5 flies
Extended Fig. 9a	one-way ANOVA followed by Tukey's post hoc test	Light response of AMA neurons	Depolarization under current clamp<br>WT (n = 6 from 6 flies) vs. CHO (n = 6 from 6 flies), P = 0.1513<br>WT (n = 6 from 6 flies) vs. CO (n = 6 from 6 flies), P = 0.6319<br>CO (n = 6 from 6 flies) vs. CHO (n = 6 from 6 flies), P = 0.6789<br><br>Inward current under voltage clamp<br>WT (n = 15 from 6 flies) vs. CHO (n = 6 from 6 flies), P = 0.9909<br>WT (n = 15 from 6 flies) vs. CO (n = 6 from 6 flies), P = 0.5688<br>CO (n = 6 from 6 flies) vs. CHO (n = 6 from 6 flies), P = 0.6463
Extended Fig. 9b	one-way ANOVA followed by Tukey's post hoc test	AMA neurons	Peak response before, during and after CIM application (n = 6 from 6 flies):<br>Before vs. during: P = 0.7662<br>During vs. after, P = 0.9270<br>Before vs. after, P = 0.5442<br><br>Steady response before, during and after CIM application (n = 6 from 6 flies):<br>Before vs. during: P = 0<br>During vs. after, P = 0<br>Before vs. after, P = 0.0974
Extended Fig. 9c	one-way ANOVA followed by Tukey's post hoc test	Light response of AMA neurons	Peak response under current clamp:<br>R8>norpA rescue (n = 6 from 6 flies) vs. R8>norpA rescue; CO (n = 6 from 6 flies), P = 0.3538<br>R8>norpA rescue (n = 6 from 6 flies) vs. R8>norpA rescue; CHO (n = 8 from 6 flies), P = 0.5171<br>R8>norpA rescue;CO (n = 6 from 6 flies) vs. R8>norpA rescue; CHO (n = 8 from 6 flies), P = 0.9726<br>Steady response under current clamp:<br>R8>norpA rescue (n = 6 from 6 flies) vs. R8>norpA rescue; CO (n = 6 from 6 flies), P = 0.7552<br>R8>norpA rescue (n = 6 from 6 flies) vs. R8>norpA rescue; CHO (n = 8 from 6 flies), P = 1.2233E-5.<br>R8>norpA rescue;CO (n = 6 from 6 flies) vs. R8>norpA rescue; CHO (n = 8 from 6 flies), P = 1.4048E-6.<br><br>Peak response under voltage clamp:<br>R8>norpA rescue (n = 6 from 6 flies) vs. R8>norpA rescue; CO (n = 8 from 6 flies), P = 0.5774<br>R8>norpA rescue (n = 6 from 6 flies) vs. R8>norpA rescue; CHO (n = 8 from 6 flies), P = 0.1858<br>R8>norpA rescue;CO (n = 6 from 6 flies) vs. R8>norpA rescue; CHO (n = 8 from 6 flies), P = 0.0164<br>Steady response under voltage clamp:<br>R8>norpA rescue (n = 6 from 6 flies) vs. R8>norpA rescue; CO (n = 8 from 6 flies), P = 0.1698<br>R8>norpA rescue (n = 6 from 6 flies) vs. R8>norpA rescue; CHO (n = 8 from 6 flies), P = 6.0293E-8.<br>R8>norpA rescue;CO (n = 6 from 6 flies) vs. R8>norpA rescue; CHO (n = 8 from 6 flies), P = 1.2942E-6.<br>Spike firing rate:<br>R8>norpA rescue (n = 5 from 5 flies)<br>R8>norpA rescue; CO (n = 5 from 5 flies)<br>R8>norpA rescue; CHO (n = 6 from 5 flies)
Extended Fig. 9e	Two-tailed Mann-	Calcium response of R8 terminal	Peak response, WT (n = 59 from 6 flies) vs. CHO (n = 58 from 5 flies), P = 0.0002<br>Steady response, WT (n = 59 from 6 flies) vs. CHO (n = 58 from 5 flies), P = 6.2870E-6

	Whitney tests		
Extended Fig. 9f		Light response of AMA neurons in saline solutions with different Ca^{2+} concentratio	Depolarization induced by dim and bright light under different calcium concentration, in the presence of CIM or MCA, n = 6
Extended Fig. 10b			l-LNv (n = 6 from 5 flies)<br>s-LNv (n = 6 from 6 flies)<br>ITP-LNd (n = 6 from 6 flies)<br>5th s-LNv (n = 6 from 6 flies)

Supplementary Table 3 | Fly strains used in this paper

Fly Strains	Source	Identifier
	**Gal4**	
DvPdf-Gal4 (II)	From P. Emery	N/A
GMR-Gal4 (II)	From P. Emery	N/A
GMR24C08-Gal4 (attp2)	Bloomington Stock Center	#48050
L1 spGal4: SS00691	From Janelia Research Campus	N/A
Tm5 spGal4: ort-Gal4.C1a.DBD; dVP16AD[ET18k]	Bloomington Stock Center	#56524
Tm9 spGal4: SS00307	From Janelia Research Campus	N/A
Tm20 spGal4: ort-Gal4.C1a.DBD, ET.VP16.AD [tou9A30]	Bloomington Stock Center	#56521
Tm20 spGal4: SS00355	From Janelia Research Campus	N/A
aMe12 spGal4: SS01050	From Janelia Research Campus	N/A
HDC-Gal4 (II)	From Y. Rao	N/A
Rh1-Gal4 (II)	Bloomington Stock Center	#8692
Rh3-Gal4 (II)	Bloomington Stock Center	#7457
Rh4-Gal4 (II)	Bloomington Stock Center	#8627
Rh5-Gal4 (II)	Bloomington Stock Center	#7458
Rh6-Gal4 (II)	Bloomington Stock Center	#7459
Rh6-Gal4 (III)	Bloomington Stock Center	#7464
VT037867-Gal4 (attp2)	Vienna Drosophila Resource Center	#v203797
	**LexA**	
DvPdf-LexA (attp2)	our own lab	N/A
DvPdf-LexA (attp40)	our own lab	N/A
GMR27G06-LexA (attp40)	Bloomington Stock Center	#54779
GMR54D11-LexA (attp2)	This study	
GMR-LexA (II)	From Y. Rao	N/A
ort-LexA (III)	From Y. Rao	N/A
Rh5-LexA (II)	From Y. Rao	N/A
Rh6-LexA (attp40)	From T. Suzuki	N/A
vGAT-LexA (II)	From Y. Rao	N/A
VT037867-LexA (attp40)	our own lab	N/A
	**QS**	
ort-QS (VK00005)	This paper	N/A
	**FLP**	
ChAT-FLP (III)	From Y. Rao	N/A
TH-FLP (III)	From Y. Rao	N/A
Trh-FLP (III)	From Y. Rao	N/A

vGlut-FLP (II)	From Y. Rao	N/A
**UAS**		
UAS-Cas9.P2 (attp40)	Bloomington Stock Center	#58985
UAS-ChAT sgRNA (attp5)	From Y. Rao	N/A
UAS-Hdc sgRNA (attp5)	From Y. Rao	N/A
UAS-VAChT sgRNA (attp5)	From Y. Rao	N/A
UAS-mSPA-GFP (III)	From Y. Rao	N/A
UAS-CsChrimson (attp40)	Bloomington Stock Center	#55135
UAS-FRT-STOP-FRT-mCD8-GFP (II)	From C. Potter	N/A
UAS-FRT-STOP-FRT-mCD8-GFP (III)	From C. Potter	N/A
UAS-GCaMP6m (attp40)	Bloomington Stock Center	#42748
UAS-GCaMP6m (VK00005)	Bloomington Stock Center	#42750
UAS-FLP(II)	Bloomington Stock Center	#4539
UAS-mCD8-GFP (II)	From C. Potter	N/A
UAS-mCD8-GFP (III)	From C. Potter	N/A
UAS-norpA.K (II)	Bloomington Stock Center	#26267
UAS-norpA.K (III)	Bloomington Stock Center	#26273
UAS-HisCl1(II)	From F. Rouyer	N/A
UAS-ort (II)	From F. Rouyer	N/A
UAS-post-GFP$^{1-10}$ (attp2)	From S. Stowers	N/A
UAS-TNTE (II)	Bloomington Stock Center	#28837
**LexAop**		
LexAop2-GCaMP6f (attp5)	Bloomington Stock Center	#44277
LexAop2-jGCaMP7s	Bloomington Stock Center	#80913
LexAop2-GCaMP6m (VK00005)	Bloomington Stock Center	#44276
LexAop-tdTomato (attp5)	From Y. Rao	N/A
LexAop-norpA (attp5)	From Y. Rao	N/A
LexAop-post-GFP^{11} (VK00005)	This paper	N/A
LexAop2-GFP (attp5)	From Y. Rao	N/A
LexAop2-GFP (attp2)	Bloomington Stock Center	#32209
**Mutant**		
cry^{02}	From T. Yoshii	N/A
cry^{02}, HisCl1^{134}, ort^{1}	From F. Rouyer	N/A
Hdc^{JK910}	Bloomington Stock Center	#64203
ort^{1}	From Y. Rao	N/A
HisCl^{134}, ort^{1}	From Y. Rao	N/A

norpA^{P41}	From P. Emery	N/A
Rh5^{2}	From F. Rouyer	N/A
ninaE^{I17}	Kyoto stock center	#109599
Rh6^{1}	Kyoto stock center	#109600
Rh6^{1}*, HisCl*^{134}*, ort*^{1}	From F. Rouyer	N/A
shakB^{2}	From J.M. Blagburn	N/A
**Others**		
MCFO-1: pBPhsFlp2::PEST (attP3); pJFRC201-10XUAS-FRT-STOP-FRT-myr::smGFP-HA (VK0005), pJFRC240-10XUAS-FRT-STOP-FRT-myr::smGFP-V5-THS-10XUAS-FRT-STOP-FRT-myr::smGFP-FLAG (su(Hw)attP1)hsFLP(attP3);; HA_V5_FLAG	Bloomington Stock Center	#64085
BAcTrace: LexAop2-Syb::GFP-P10 (VK37) LexAop-QF2::SNAP25::HIVNES::Syntaxin (VK18); UAS-B3Recombinase (attP2) UAS<B3STOP<BoNT/A (VK5) UAS<B3STOP<BoNT/A(VK27) QUAS-mtdTomato::HA	From G. Jefferis	N/A
GRASP: UAS-GFP$^{1\text{-}10}$*, LexAop-GFP*^{11}	From Y. Rao	N/A
trans-Tango: UAS-myrGFP.QUAS-mtdTomato-3×HA(attp8); trans-Tango (attp40)	Bloomington Stock Center	#77124
UAS-FRT-STOP-FRT-CsChrimson, LexAop-FLP (II)	From C. Zhou	N/A
UAS-FRT-STOP-FRT-CsChrimson, LexAop-FLP (III)	From C. Zhou	N/A
VAChT-FRT-STOP-FRT-HA (III)	Bloomington Stock Center	#76021
GMR-RFP (II)	From Y. Rao	N/A
Rh5-eGFP (II)	Bloomington Stock Center	#8600
Rh6-eGFP (II)	Bloomington Stock Center	#7461
LexAop-GFP, UAS-RFP (I)	From C. Liu	N/A
Rh6-hid,rpr (attp5)	This paper	N/A
