# Supplementary material for: A single photoreceptor splits perception and entrainment by cotransmission
Source: Nature. 2023 Oct 25;623(7987):562–70. doi: 10.1038/s41586-023-06681-6 (PMC10651484; doi:10.1038/s41586-023-06681-6)
Supplement: Supplementary file 2 — Reporting Summary [file 41586_2023_6681_MOESM2_ESM.pdf]

nature portfolio

Corresponding author(s): Dong-Gen Luo

Last updated by author(s): Sep 10, 2023

Reporting Summary

Nature Portfolio wishes to improve the reproducibility of the work that we publish. This form provides structure for consistency and transparency in reporting. For further information on Nature Portfolio policies, see our Editorial Policies and the Editorial Policy Checklist.

Statistics

For all statistical analyses, confirm that the following items are present in the figure legend, table legend, main text, or Methods section.

n/a	Confirmed	
☐	☒	The exact sample size (*n*) for each experimental group/condition, given as a discrete number and unit of measurement
☐	☒	A statement on whether measurements were taken from distinct samples or whether the same sample was measured repeatedly
☐	☒	The statistical test(s) used AND whether they are one- or two-sided<br>*Only common tests should be described solely by name; describe more complex techniques in the Methods section.*
☒	☐	A description of all covariates tested
☐	☒	A description of any assumptions or corrections, such as tests of normality and adjustment for multiple comparisons
☐	☒	A full description of the statistical parameters including central tendency (e.g. means) or other basic estimates (e.g. regression coefficient) AND variation (e.g. standard deviation) or associated estimates of uncertainty (e.g. confidence intervals)
☒	☐	For null hypothesis testing, the test statistic (e.g. *F*, *t*, *r*) with confidence intervals, effect sizes, degrees of freedom and *P* value noted<br>*Give P values as exact values whenever suitable.*
☒	☐	For Bayesian analysis, information on the choice of priors and Markov chain Monte Carlo settings
☒	☐	For hierarchical and complex designs, identification of the appropriate level for tests and full reporting of outcomes
☒	☐	Estimates of effect sizes (e.g. Cohen's *d*, Pearson's *r*), indicating how they were calculated

Our web collection on statistics for biologists contains articles on many of the points above.

Software and code

Policy information about availability of computer code

Data collection	Patch-clamp recording data were acquired using Clampex 10.6 (https://www.moleculardevices.com/).<br>Imaging data were acquired using NIS-Element (https://www.microscope.healthcare.nikon.com/).<br>Locomotion activity data were acquired using DAM2 system (https://trikinetics.com/).
Data analysis	Data analysis were performed using Matlab 2020b (https://www.mathworks.com/products/matlab.html), Origin 2022(https://www.originlab.com/), Fiji (https://fiji.sc/) and GraphPad Prism 9 (https://www.graphpad.com/scientific-software/prism/).

For manuscripts utilizing custom algorithms or software that are central to the research but not yet described in published literature, software must be made available to editors and reviewers. We strongly encourage code deposition in a community repository (e.g. GitHub). See the Nature Portfolio guidelines for submitting code & software for further information.

Data

Policy information about availability of data

All manuscripts must include a data availability statement. This statement should provide the following information, where applicable:

- Accession codes, unique identifiers, or web links for publicly available datasets
- A description of any restrictions on data availability
- For clinical datasets or third party data, please ensure that the statement adheres to our policy

Behavioral, electrophysiological, morphological raw data and additional information required to reanalyze the data reported in this paper are available from the corresponding upon request.

Human research participants

Policy information about studies involving human research participants and Sex and Gender in Research.

Reporting on sex and gender	n/a
Population characteristics	n/a
Recruitment	n/a
Ethics oversight	n/a

Note that full information on the approval of the study protocol must also be provided in the manuscript.

Field-specific reporting

Please select the one below that is the best fit for your research. If you are not sure, read the appropriate sections before making your selection.

☒ Life sciences ☐ Behavioural & social sciences ☐ Ecological, evolutionary & environmental sciences

For a reference copy of the document with all sections, see nature.com/documents/nr-reporting-summary-flat.pdf

Life sciences study design

All studies must disclose on these points even when the disclosure is negative.

Sample size	No statistical methods were used to determine sample size. Sample sizes were based on the prior studies using similar assays.
Data exclusions	No data was excluded from analysis.
Replication	See Supplementary Table 2 for repeats of cells and animals.
Randomization	Experiments were not randomized.
Blinding	The investigators were not blind to fly genotypes, as the fly crosses were done by the investigators who also performed the experiments.

Reporting for specific materials, systems and methods

We require information from authors about some types of materials, experimental systems and methods used in many studies. Here, indicate whether each material, system or method listed is relevant to your study. If you are not sure if a list item applies to your research, read the appropriate section before selecting a response.

Materials & experimental systems

n/a	Involved in the study
☐	☒ Antibodies
☒	☐ Eukaryotic cell lines
☒	☐ Palaeontology and archaeology
☐	☒ Animals and other organisms
☒	☐ Clinical data
☒	☐ Dual use research of concern

Methods

n/a	Involved in the study
☒	☐ ChIP-seq
☒	☐ Flow cytometry
☒	☐ MRI-based neuroimaging

Antibodies

Antibodies used	Primary antibodies: rat anti-LOVIT,from T. Wang; rabbit anti-TIM, from our own lab; rabbit anti-HA, 3724, Cell Signaling Technology; mouse anti-nc82, DSHB; mouse anti-V5 DyLight 549, MCA2894D549GA, Bio-Rad; mouse anti-V5 DyLight 647, MCA1360A647, Bio-Rad.<br>Secondary antibodies:<br>goat anti-rat Alexa Fluor 568, ab175710, Abcam; goat anti-rabbit Alexa Fluor 488, A27034, Thermo Fisher; goat anti-mouse Alexa Fluor 568, A11004, Thermo Fisher)
Validation	all of the antibodies used in this study were commercial or had been published in previous studies. validation statements can be found on the manufacturer's website.

Animals and other research organisms

Policy information about studies involving animals; ARRIVE guidelines recommended for reporting animal research, and Sex and Gender in Research

Laboratory animals	See Supplementary Table 1 and Table3 for fly strains used in this study.
Wild animals	No wild animals were used in this study.
Reporting on sex	Animals of either sex were used.
Field-collected samples	No field collected samples were used in this study.
Ethics oversight	This study did not require ethical approval.

Note that full information on the approval of the study protocol must also be provided in the manuscript.
